# Longitudinal trajectories of polypharmacy in older people, and their association with the risk of mortality: a joint latent class model analysis of real-world data from the UK and the Netherlands

**DOI:** 10.1093/ageing/afaf233

**Published:** 2025-08-20

**Authors:** Leena Elhussein, Ross D Williams, Wai Yi Man, Edward Burn, Antonella Delmestri, Victoria Y Strauss, Daniel Prieto-Alhambra

**Affiliations:** Nuffield Department of Orthopaedics, Rheumatology and Musculoskeletal Sciences, University of Oxford, Oxford, UK; Department of Medical Informatics, Erasmus University Medical Center, Rotterdam, Netherlands; Nuffield Department of Orthopaedics, Rheumatology and Musculoskeletal Sciences, University of Oxford, Oxford, UK; Nuffield Department of Orthopaedics, Rheumatology and Musculoskeletal Sciences, University of Oxford, Oxford, UK; Nuffield Department of Orthopaedics, Rheumatology and Musculoskeletal Sciences, University of Oxford, Oxford, UK; Nuffield Department of Orthopaedics, Rheumatology and Musculoskeletal Sciences, University of Oxford, Oxford, UK; Nuffield Department of Orthopaedics, Rheumatology and Musculoskeletal Sciences, University of Oxford, Oxford, UK

**Keywords:** joint latent class models, older people, polypharmacy, longitudinal, mortality

## Abstract

**Objective:**

Polypharmacy is the use of multiple drugs. Many definitions have been established for polypharmacy, often cross-sectionally, despite it naturally changing over time. In this study, we aimed to identify clusters of older people with distinct polypharmacy trajectories over time and associated mortality risks. We then characterised the identified clusters and assessed their generalisability in two external databases.

**Methods:**

Data were extracted from three primary care databases: the UK Clinical Practice Research Datalink (CPRD) GOLD, CPRD Aurum and the Dutch Integrated Primary Care Information (IPCI). People aged ≥65 on 1 January 2015 were included. Polypharmacy, defined as the cumulative number of prescribed ingredients, was calculated at baseline and at the end of each subsequent follow-up year (2015–19). We applied joint latent class modelling, which divides the population into clusters with different trajectories and associated mortality risks. The model was trained in GOLD and validated in Aurum and IPCI.

**Results:**

Four clusters were identified and characterised based on polypharmacy baseline and rate of progression: low-steady, intermediate-slow/increasing, intermediate-fast/increasing and high-decreasing. The high-decreasing cluster had the highest average baseline polypharmacy (intercept = 23.4) and prevalence of non-cancer chronic comorbidities, whilst the intermediate-fast/increasing had the steepest polypharmacy rate of progression per year (slope = 6.4), highest baseline and cumulative incidence of cancer, and worst survival outcome. Good validation was found in Aurum and IPCI.

**Conclusion:**

High baseline levels and increasing levels of polypharmacy were associated with an increased mortality risk in older people. The clusters identified in this study were externally validated in two European databases, confirming their robustness and generalisability.

## Key Points

- Polypharmacy is often described as a cross-sectional measure even though it naturally fluctuates over time.
- We identified four distinct groups of older people from UK primary care data based on their polypharmacy variation over time.
- The groups were: low-steady, intermediate-slow/increasing, intermediate-fast/increasing and high-decreasing.
- The intermediate-fast/increasing group had the highest mortality risk.
- The groups identified were externally validated in UK and Dutch databases, indicating their generalisability.

## Introduction

Polypharmacy is the use of multiple drugs either concurrently or cumulatively. It is often defined based on a cross-sectional value, e.g. use of ≥5 or ≥10 in a given period [1]. Along with polypharmacy, problems like poor adherence, complex medication regimens and inappropriate polypharmacy arise [2–4]. Furthermore, polypharmacy is associated with multiple adverse events, such as drug–drug interactions, adverse drug reactions, hospitalisation and mortality [1, 5–8].

Studies investigating the short- and long-term effects of polypharmacy have found an association between polypharmacy and the risk of death, falls and multimorbidity [9–11]. Richardson *et al*. found that using ≥5 drugs increased the risk of 2-year mortality by ≥80% for UK patients aged over 65 [9].

Previous research has described variation over time [12–14]. A Dutch study found that besides age and baseline number of drugs, chronic morbidities such as diabetes and hypertension were predictive of polypharmacy after a 4-year follow-up [12]. Others confirmed that polypharmacy increases more rapidly for patients with multimorbidity [15], patients leaving the study early [16] or during the year before death [13]. However, there is still a gap in identifying polypharmacy changing over time whilst simultaneously accounting for censored data and heterogeneity in older populations.

We aimed to identify and characterise sub-groups (clusters) of older people with distinct polypharmacy trajectories over 5 years, and to assess the association between these clusters and their risk of death. The clusters identified were validated in two independent databases.

## Methods

### Summary of design

We assessed the change in polypharmacy over 5 years by conducting a longitudinal cohort analysis. We identified random samples of UK and Dutch older people from primary care databases. Polypharmacy was calculated annually during the follow-up period. We applied joint latent class models to identify clusters with distinct polypharmacy patterns over time and assessed their association with all-cause mortality. The identified clusters were characterised, and the model validated in two external datasets.

### Data source

Data were extracted from three primary care databases: UK Clinical Practice Research Datalink (CPRD) GOLD, CPRD Aurum and Dutch Integrated Primary Care Information (IPCI). These databases record the reasons for all consultations in primary care but also include information on laboratory tests, other assessments, diagnoses, immunisations and prescriptions [17–19]. They were representative of their respective populations. The three databases were mapped to the Observational Medical Outcomes Partnership Common Data Model (OMOP-CDM). OMOP-CDM standardises different patient-level observational data into a common format having the same structure, and vocabularies [20]. CPRD GOLD and CPRD Aurum will be referred to as ‘GOLD’ and ‘Aurum’. Additional information about the three databases is described in Appendix 1.

### Population

We included random samples of older people aged ≥65, alive on 1 January 2015 (start date), and a minimum 365 day look back. Duplicate patients belonging to both GOLD and Aurum were removed from the Aurum sample.

Follow-up period was observed from 1 January 2015 to 31 December 2019. The choice of this end date avoided different trends potentially caused by COVID-19. Patients exited the study at the earliest of: practice last collection date, patient transfer-out of practice date, death date [21] or study end date.

### Exposure and outcome

Exposure was polypharmacy, defined as the absolute number of ingredients. Polypharmacy was calculated at baseline, and at the end of each follow-up year. This simple cumulative measure of polypharmacy is the most used definition. It allows for a comprehensive assessment of medication use whilst also allowing for the detection of intermittent medications and chronic diseases’ treatment changes [22, 23], which could be offset by more frequent measures [24]. Table S1 in Appendix 1 explains the difference between prescriptions described as substances in the source CPRD data (British National Formulary) vs. ingredients in the OMOP-CDM.

The outcome of interest was all-cause mortality, defined as death recorded in the databases during follow-up [19, 25].

### Statistical analyses

#### Descriptive analysis

The baseline demographics were extracted and compared between the three cohorts.

#### Joint latent class modelling

To identify clusters of distinct patterns of polypharmacy change and associated mortality risks, we applied joint latent class modelling (JLCM). JLCM is a longitudinal method to describe trajectories of markers/exposures over time and their association with the risk of an adverse event. It assumes a heterogeneous population and uses latent class modelling to divide the population into homogeneous classes (clusters) with similar trajectories and risk of adverse events [26, 27].

Age and sex were accounted for in the derivation of clusters in both longitudinal and time-to-event models. Two- to six-cluster models were fitted. The optimum number of clusters was based on model convergence, Bayesian information criterion (BIC), clinical plausibility and minimum cluster size (≥1%).

#### Characterisation of the resulting clusters

To characterise the resulting clusters, comorbidities and drug use were compared across the clusters at baseline and last observation. These included Charlson Comorbidity Index (CCI), drug classes—according to the World Health Organisation’s Anatomical Therapeutic Chemical (ATC) Classification level 1; pharmacological or therapeutic groups, individual conditions and drug exposures.

#### Model validation

We assessed the performance of the best-fit cluster model (derived from GOLD) in Aurum and IPCI. Good validation was defined as average posterior probabilities of belonging to the pre-defined clusters being ≥0.7 for at least 50% of each cluster. The threshold of 0.7 indicates a clear classification of people into clusters [28]. Additionally, we compared the mortality risks and characteristics of the clusters identified in the validation sample to the original model.

We used R (Version 4.2.3) for analyses. R packages ‘lcmm’ version 2.0.2 https://cran.r-project.org/web/packages/lcmm/index.html, and ‘Patient Profiles’ version 0.4.0 https://cran.r-project.org/web/packages/PatientProfiles/index.html were used for JLCM, and characterisation of resultant clusters.

## Results

### Model training in GOLD

#### Study population

We included 299 859 out of 1 028 458 eligible GOLD patients. Mean age was 75.3 (SD 7.9) with 45.4% male patients. At baseline, the median number of ingredients taken the prior year was 8 [IQR 4–13] and mean CCI was 1.2 (SD 1.5) (see Table 1).

**Table 1:** Baseline characteristics by cluster and for the overall population in GOLD, Aurum and IPCI.

	Intermediate-fast/increasing	Low-steady	Intermediate-slow/increasing	High-decreasing	Overall
GOLD					
*n*	3708 (1.2%)	256 923 (85.7%)	19 207 (6.4%)	20 021 (6.7%)	299 859
Sex = Male [*n* (%)]	1838 (49.6%)	117 450 (45.7%)	8745 (45.5%)	7987 (39.9%)	136 020 (45.4%)
Age [mean (SD)]	76.73 (7.56)	75.01 (7.90)	76.77 (7.85)	76.86 (7.88)	75.26 (7.91)
Number of ingredients [median (IQR)]^a^	11 [6, 16]	7 [3, 11]	14 [10, 19]	26 [22, 30]	8 [4, 13]
CCI [mean (SD)]	1.95 (1.92)	1.03 (1.41)	1.83 (1.80)	2.32 (1.99)	1.18 (1.54)
Aurum					
*n*	3396 (1.3%)	225 383 (85.0%)	20 639 (7.8%)	15 683 (5.9%)	265 101
Sex = Male [*n* (%)]	1760 (51.8%)	103 242 (45.8%)	9607 (46.5%)	6275 (40.0%)	120 884 (45.6%)
Age [mean (SD)]	76.98 (7.52)	74.96 (7.84)	77.20 (7.95)	77.36 (7.96)	75.3 (7.90)
Number of ingredients [median (IQR)]^a^	11 [7, 16]	7 [3, 11]	14 [9, 19]	25 [22, 30]	8 [4, 13]
CCI [mean (SD)]	2.48 (2.10)	1.26 (1.54)	2.31 (1.96)	2.83 (2.10)	1.45 (1.69)
IPCI					
n	1429 (1.0%)	125 835 (90.3%)	8268 (5.9%)	3775 (2.7%)	139 307
Sex = Male [*n* (%)]	814 (57.0%)	56 789 (45.1%)	4138 (50.0%)	1565 (41.5%)	63 306 (45.4%)
Age [mean (SD)]	75.29 (6.96)	74.10 (7.31)	75.09 (7.14)	75.29 (7.13)	74.21 (7.30)
Number of ingredients [median (IQR)]^a^	9 [5, 14]	6 [3, 10]	12 [7, 16]	24 [21, 28]	6 [3, 10]
CCI [mean (SD)]	2.17 (1.71)	1.16 (1.39)	1.99 (1.67)	2.64 (1.90)	1.26 (1.46)

^a^In the year prior to start.

#### Joint latent class modelling

The GOLD population was followed up for 4.1 years [IQR 1.4–5.0], with 38 031 (12.7%) patients dying during the study period.

**Figure 1:**
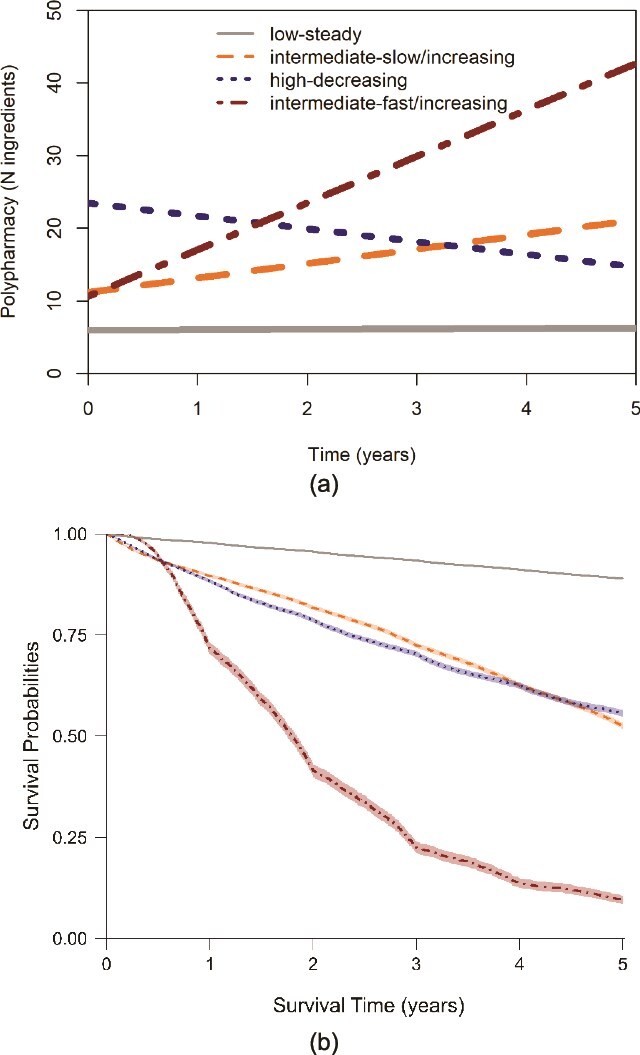
(a) Intercepts and slopes of ingredients and (b) survival probabilities of each cluster in GOLD.

The four-cluster model was the optimum model after assessing BIC, convergence, smallest cluster size and clinical plausibility (Appendix 2; Tables S2 and S3). The four clusters identified were: low-steady (85.7%, intercept = 5.97, slope = 0.07), intermediate-slow/increasing (6.4%, intercept = 11.22, slope = 1.98), intermediate-fast/increasing (1.2%, intercept = 10.70, slope = 6.40) and high-decreasing (6.7%, intercept = 23.35, slope = −1.75). Taking the low-steady class (7.6% deaths) as reference, the intermediate-fast/increasing cluster had the highest risk of mortality, with an adjusted hazard ratio (HR) of 20.5 (95%CI 19.9, 21.2); and 83.8% of patients in this cluster died over the 5-year follow-up period. The second and third highest mortality were the intermediate-slow/increasing and high-decreasing clusters, with HR of 4.95 (95%CI 4.84, 5.07) and 4.64 (95%CI 4.58, 4.73). Figure 1 describes these clusters in terms of progression over time and survival probabilities. Median follow-up, yearly deaths and polypharmacy are reported in Appendix 2; Table S4. Compared to the low-steady, the other clusters had a slightly higher mean age (75.0 vs. 76.7–76.9) at baseline (Table 1).

#### Characterisation of the identified clusters

##### Comorbidities

The low-steady cluster had the lowest CCI at baseline and at last observation [mean 1.0 (SD 1.4), Δ = 0.4]. Conversely, the high-decreasing cluster had the highest CCI at baseline but a slow increase over time [mean 2.3 (SD 2.0), Δ = 0.6]. The intermediate-high/increasing and intermediate-slow/increasing clusters had slightly lower baseline index but bigger increases in CCI by the last observation; [mean 2.0 (SD 1.9), Δ = 1.5] and [mean 1.8 (SD 1.8), Δ = 1.1] (Table 2).

**Table 2:** Individual morbidities at baseline and the last observation (including Charlson morbidities) for each cluster and the overall population in GOLD.

	At baseline					At the last observation				
	Intermediate-fast/increasing	Low-steady	Intermediate-slow/increasing	High-decreasing	Overall	Intermediate-fast/increasing	Low-steady	Intermediate-slow/increasing	High-decreasing	Overall
	*N* = 3708	*N* = 256 923	*N* = 19 207	*N* = 20 021	*N* = 299 859	*N* = 3708	*N* = 256 923	*N* = 19 207	*N* = 20 021	*N* = 299 859
Mean (SD)										
CCI	1.95 (1.92)	1.03 (1.41)	1.83 (1.80)	2.32 (1.99)	1.18 (1.54)	3.46 (2.54)	1.39 (1.66)	2.95 (2.28)	2.90 (2.20)	1.61 (1.84)
*n* (%)										
Myocardial infarction	190 (5.1)	7944 (3.1)	1170 (6.1)	1744 (8.7)	11 048 (3.7)	331 (8.9)	10 518 (4.1)	1949 (10.1)	2119 (10.6)	14 917 (5.0)
Congestive heart failure	204 (5.5)	5491 (2.1)	1187 (6.2)	1875 (9.4)	8757 (2.9)	422 (11.4)	9039 (3.5)	2565 (13.4)	2640 (13.2)	14 666 (4.9)
Peripheral vascular disease	168 (4.5)	4051 (1.6)	737 (3.8)	1023 (5.1)	5979 (2.0)	217 (5.9)	5489 (2.1)	1143 (6.0)	1249 (6.2)	8098 (2.7)
Cerebrovascular disease	431 (11.6)	14 468 (5.6)	2023 (10.5)	2715 (13.6)	19 637 (6.5)	603 (16.3)	20 598 (8.0)	3121 (16.2)	3386 (16.9)	27 708 (9.2)
Dementia	251 (6.8)	7166 (2.8)	1029 (5.4)	1165 (5.8)	9611 (3.2)	421 (11.4)	14 297 (5.6)	2227 (11.6)	2102 (10.5)	19 047 (6.4)
Chronic obstructive pulmonary disease	808 (21.8)	31 383 (12.2)	4645 (24.2)	6544 (32.7)	43 380 (14.5)	986 (26.6)	38 033 (14.8)	6067 (31.6)	7334 (36.6)	52 420 (17.5)
Rheumatologic disease	156 (4.2)	8159 (3.2)	998 (5.2)	1657 (8.3)	10 970 (3.7)	195 (5.3)	10 475 (4.1)	1534 (8.0)	1891 (9.4)	14 095 (4.7)
Peptic ulcer disease	115 (3.1)	5174 (2.0)	625 (3.3)	925 (4.6)	6839 (2.3)	165 (4.4)	6332 (2.5)	896 (4.7)	1090 (5.4)	8483 (2.8)
Mild liver disease	32 (0.9)	685 (0.3)	130 (0.7)	189 (0.9)	1036 (0.3)	63 (1.7)	1024 (0.4)	235 (1.2)	251 (1.3)	1573 (0.5)
Diabetes with chronic complications	381 (10.3)	11 909 (4.6)	2140 (11.1)	3234 (16.2)	17 664 (5.9)	486 (13.1)	15 964 (6.2)	3046 (15.9)	3990 (19.9)	23 486 (7.8)
Hemiplegia or paraplegia	9 (0.2)	208 (0.1)	44 (0.2)	54 (0.3)	315 (0.1)	13 (0.4)	242 (0.1)	64 (0.3)	61 (0.3)	380 (0.1)
Renal disease	895 (24.1)	41 212 (16.0)	5146 (26.8)	6466 (32.3)	53 719 (17.9)	1163 (31.4)	51 576 (20.1)	6958 (36.2)	7730 (38.6)	67 427 (22.5)
Any malignancy	960 (25.9)	34 856 (13.6)	3561 (18.5)	4015 (20.1)	43 392 (14.5)	1991 (53.7)	48 318 (18.8)	6427 (33.5)	5190 (25.9)	61 926 (20.7)
Moderate to severe liver disease	18 (0.5)	184 (0.1)	55 (0.3)	87 (0.4)	344 (0.1)	42 (1.1)	326 (0.1)	122 (0.6)	125 (0.6)	615 (0.2)
Metastatic solid tumour	55 (1.5)	454 (0.2)	105 (0.5)	141 (0.7)	755 (0.3)	333 (9.0)	1262 (0.5)	592 (3.1)	279 (1.4)	2466 (0.8)
AIDS	1 (0)	18 (0)	1 (0)	0 (0)	20 (0)	1 (0)	21 (0)	2 (0)	1 (0)	25 (0)
Hypertension	1254 (33.8)	82 262 (32.0)	6965 (36.3)	7683 (38.4)	98 164 (32.7)	1334 (36.0)	91 691 (35.7)	7835 (40.8)	8126 (40.6)	108 986 (36.4)
Heart failure	234 (6.3)	6175 (2.4)	1330 (6.9)	2036 (10.2)	9775 (3.3)	458 (12.4)	10 071 (3.9)	2769 (14.4)	2836 (14.2)	16 134 (5.4)
Osteoporosis	309 (8.3)	14 311 (5.6)	1847 (9.6)	2852 (14.3)	19 319 (6.4)	435 (11.7)	18 594 (7.2)	2875 (15.0)	3378 (16.9)	25 282 (8.4)
Gastroesophageal reflux disease	120 (3.2)	8460 (3.3)	935 (4.9)	1397 (7.0)	10 912 (3.6)	161 (4.3)	10 466 (4.1)	1250 (6.5)	1618 (8.1)	13 495 (4.5)
Chronic kidney disease	882 (23.8)	40 772 (15.9)	5100 (26.6)	6390 (31.9)	53 144 (17.7)	1062 (28.6)	49 666 (19.3)	6501 (33.9)	7385 (36.9)	64 614 (21.6)
Venous thromboembolism	233 (6.3)	11 996 (4.7)	1329 (6.9)	1972 (9.9)	15 530 (5.2)	463 (12.5)	15 397 (6.0)	2171 (11.3)	2464 (12.3)	20 495 (6.8)
Hypothyroidism	288 (7.8)	16 937 (6.6)	1792 (9.3)	2267 (11.3)	21 284 (7.1)	335 (9.0)	19 153 (7.5)	2127 (11.1)	2489 (12.4)	24 104 (8.0)
Stroke	218 (5.9)	6784 (2.6)	1058 (5.5)	1354 (6.8)	9414 (3.1)	331 (8.9)	9996 (3.9)	1725 (9.0)	1741 (8.7)	13 793 (4.6)
Anxiety	519 (14.0)	29 632 (11.5)	2980 (15.5)	4692 (23.4)	37 823 (12.6)	648 (17.5)	34 024 (13.2)	3788 (19.7)	5275 (26.4)	43 735 (14.6)
Asthma	408 (11.0)	19 425 (7.6)	2716 (14.1)	4187 (20.9)	26 736 (8.9)	456 (12.3)	21 893 (8.5)	3214 (16.7)	4546 (22.7)	30 109 (10.0)
Pneumonia	182 (4.9)	4416 (1.7)	736 (3.8)	1447 (7.2)	6781 (2.3)	508 (13.7)	8437 (3.3)	2225 (11.6)	2498 (12.5)	13 668 (4.6)
Diabetes	630 (17.0)	27 625 (10.8)	3755 (19.6)	4922 (24.6)	36 932 (12.3)	772 (20.8)	33 544 (13.1)	4681 (24.4)	5551 (27.7)	44 548 (14.9)
Inflammatory bowel disease	38 (1.0)	1672 (0.7)	190 (1.0)	273 (1.4)	2173 (0.7)	44 (1.2)	1889 (0.7)	243 (1.3)	296 (1.5)	2472 (0.8)
Depressive disorder	499 (13.5)	25 187 (9.8)	2848 (14.8)	4571 (22.8)	33 105 (11.0)	603 (16.3)	28 021 (10.9)	3481 (18.1)	5034 (25.1)	37 139 (12.4)

The main characteristic of the intermediate/fast-increasing cluster was the high prevalence of cancer: 25.9% of the patients in this cluster had malignancy/ies at baseline, rising to 53.7% at the last observation (Δ = 27.8%). It was followed by the intermediate-slow/increasing cluster: 18.5% of patients had prior malignancy diagnosis at baseline, rising to 33.5% (Δ = 15.0%) at the last observation.

The high-decreasing cluster had the highest burden of non-cancer chronic disease at baseline and at the last observation. This was observed for chronic obstructive pulmonary disease, diabetes, chronic kidney disease and osteoporosis.

The intermediate-slow/increasing cluster participants had the highest onset of most chronic diseases (excluding cancer) over the study period. For example, 26.6% had chronic kidney disease at baseline, rising to 33.9% (Δ = 7.3%) at the last observation in the intermediate-slow/increasing, compared to 31.9% at baseline rising to 36.9% (Δ = 5.0) at end of follow-up in the high-decreasing cluster.

##### Medication use

The use of drugs and drug classes followed the overall trend of each cluster, e.g. the use of drugs from the musculoskeletal system class decreased from 62.0% at baseline to 46.7% at the end of follow-up (Δ = −15.3%) for the high-decreasing cluster (Table 3).

**Table 3:** Drug use by drug classes and common drugs at baseline and the last observation for each cluster and the overall population in GOLD.

	At baseline [*n* (%)]					At the last observation [*n* (%)]				
	Intermediate-fast/increasing	Low-steady	Intermediate-slow/increasing	High-decreasing	Overall	Intermediate-fast/increasing	Low-steady	Intermediate-slow/increasing	High-decreasing	Overall
	*N* = 3708	*N* = 256 923	*N* = 19 207	*N* = 20 021	*N* = 299 859	*N* = 3708	*N* = 256 923	*N* = 19 207	*N* = 20 021	*N* = 299 859
Drug classes (ATC name)										
A: alimentary tract and metabolism	2833 (76.4)	154 318 (60.1)	16 518 (86.0)	19 935 (99.6)	193 604 (64.6)	3701 (99.8)	153 680 (59.8)	18 975 (98.8)	18 953 (94.7)	195 309 (65.1)
B: blood and blood forming organs	1924 (51.9)	86 176 (33.5)	11 498 (59.9)	15 068 (75.3)	114 666 (38.2)	2701 (72.8)	93 829 (36.5)	14 212 (74.0)	14 096 (70.4)	124 838 (41.6)
C: cardiovascular system	2767 (74.6)	167 390 (65.2)	16 098 (83.8)	18 681 (93.3)	204 936 (68.3)	3130 (84.4)	171 949 (66.9)	17 088 (89.0)	17 484 (87.3)	209 651 (69.9)
D: dermatological	1446 (39.0)	78 421 (30.5)	9376 (48.8)	15 957 (79.7)	105 200 (35.1)	2434 (65.6)	68 036 (26.5)	12 442 (64.8)	12 238 (61.1)	95 150 (31.7)
G: genito-urinary system and sex hormones	710 (19.1)	41 727 (16.2)	4724 (24.6)	7338 (36.7)	54 499 (18.2)	931 (25.1)	41 997 (16.3)	5878 (30.6)	5810 (29.0)	54 616 (18.2)
H: systemic hormonal preparations, excluding sex hormones and insulins	474 (12.8)	28 391 (11.1)	3194 (16.6)	4659 (23.3)	36 718 (12.2)	627 (16.9)	29 786 (11.6)	3904 (20.3)	4354 (21.7)	38 671 (12.9)
J: anti-infective for systemic use	1966 (53.0)	99 729 (38.8)	11 632 (60.6)	17 480 (87.3)	130 807 (43.6)	3021 (81.5)	81 383 (31.7)	14 806 (77.1)	13 138 (65.6)	112 348 (37.5)
L: antineoplastic and immunomodulation agents	305 (8.2)	10 089 (3.9)	1303 (6.8)	2006 (10.0)	13 703 (4.6)	444 (12.0)	11 468 (4.5)	1971 (10.3)	1729 (8.6)	15 612 (5.2)
M: musculoskeletal system	1281 (34.5)	73 698 (28.7)	7976 (41.5)	12 409 (62.0)	95 364 (31.8)	1764 (47.6)	64 176 (25.0)	9654 (50.3)	9348 (46.7)	84 942 (28.3)
N: nervous system	2428 (65.5)	117 985 (45.9)	14 463 (75.3)	18 800 (93.9)	153 676 (51.2)	3586 (96.7)	114 847 (44.7)	17 519 (91.2)	17 605 (87.9)	153 557 (51.2)
P: anti-parasitic products, insecticides and repellents	305 (8.2)	10 089 (3.9)	1303 (6.8)	2006 (10.0)	13 703 (4.6)	444 (12.0)	11 468 (4.5)	1971 (10.3)	1729 (8.6)	15 612 (5.2)
R: respiratory system	1826 (49.2)	81 876 (31.9)	10 847 (56.5)	15 914 (79.5)	110 463 (36.8)	2960 (79.8)	75 849 (29.5)	14 052 (73.2)	13 400 (66.9)	106 261 (35.4)
S: sensory organs	440 (11.9)	24 551 (9.6)	2787 (14.5)	5112 (25.5)	32 890 (11.0)	1338 (36.1)	25 376 (9.9)	4558 (23.7)	3936 (19.7)	35 208 (11.7)
V: various	12 (0.3)	156 (0.1)	48 (0.2)	178 (0.9)	394 (0.1)	64 (1.7)	181 (0.1)	151 (0.8)	170 (0.8)	566 (0.2)
Drugs (ATC name)										
A02 drugs for acid related disorder	2570 (69.3)	144 275 (56.2)	14 799 (77.1)	18 227 (91.0)	179 871 (60.0)	3313 (89.4)	164 943 (64.2)	17 220 (89.7)	18 803 (93.9)	204 279 (68.1)
A10A/A10B drugs used in diabetes	635 (17.1)	25 264 (9.8)	3952 (20.6)	5542 (27.7)	35 393 (11.8)	778 (21.0)	29 850 (11.6)	4731 (24.6)	5982 (29.9)	41 341 (13.8)
B01A anti-thrombotic agents	1168 (31.5)	48 833 (19.0)	6689 (34.8)	8879 (44.4)	65 569 (21.9)	1874 (50.5)	67 547 (26.3)	10 358 (53.9)	10 569 (52.8)	90 348 (30.1)
C03 diuretics	1957 (52.8)	96 627 (37.6)	11 327 (59.0)	14 192 (70.9)	124 103 (41.4)	2523 (68.0)	108 436 (42.2)	13 786 (71.8)	15 201 (75.9)	139 946 (46.7)
C07 beta blocking agents	1441 (38.9)	81 515 (31.7)	8655 (45.1)	10 383 (51.9)	101 994 (34.0)	1813 (48.9)	92 261 (35.9)	10 753 (56.0)	11 278 (56.3)	116 105 (38.7)
C08 calcium channel blockers	1598 (43.1)	89 837 (35.0)	9428 (49.1)	11 424 (57.1)	112 287 (37.5)	1794 (48.4)	104 550 (40.7)	11 039 (57.5)	12 185 (60.9)	129 568 (43.2)
C09 agents renin-angiotensin systemic	1927 (52.0)	106 426 (41.4)	11 310 (58.9)	13 568 (67.8)	133 231 (44.4)	2136 (57.6)	117 443 (45.7)	12 776 (66.5)	14 190 (70.9)	146 545 (48.9)
C10 lipid modifying agents	2237 (60.3)	126 158 (49.1)	13 104 (68.2)	15 393 (76.9)	156 892 (52.3)	2449 (66.1)	141 264 (55.9)	14 495 (75.5)	15 910 (79.5)	174 118 (58.1)
G03A hormonal contraceptives for systemic use	97 (2.6)	8904 (3.5)	581 (3.0)	806 (4.0)	10 388 (3.5)	122 (3.3)	9032 (3.5)	620 (3.2)	835 (4.2)	10 609 (3.5)
J01 antibacterial systemic	3353 (90.4)	213 020 (82.9)	18 065 (94.1)	19 782 (98.8)	254 220 (84.8)	3638 (98.1)	225 382 (87.7)	19 008 (99.0)	19 934 (99.6)	267 962 (89.4)
L01 antineoplastic agents	281 (7.6)	15 320 (6.0)	2210 (11.5)	3242 (16.2)	21 053 (7.0)	310 (8.4)	16 491 (6.4)	2478 (12.9)	3401 (17.0)	22 680 (7.6)
L04A immunosuppressant	101 (2.7)	4390 (1.7)	733 (3.8)	1238 (6.2)	6462 (2.2)	127 (3.4)	5227 (2.0)	1007 (5.2)	1385 (6.9)	7746 (2.6)
M01A/H02 anti-inflammatory and/or anti-rheumatic	2644 (71.3)	168 062 (65.4)	14 796 (77.0)	17 302 (86.4)	202 804 (67.6)	3150 (85.0)	180 436 (70.2)	16 497 (85.9)	17 900 (89.4)	217 983 (72.7)
N02A opioids	2731 (73.7)	146 030 (56.8)	15 389 (80.1)	18 238 (91.1)	182 388 (60.8)	3506 (94.55)	162 322 (63.2)	17 687 (92.1)	18 890 (94.4)	202 405 (67.5)
N03 anti-epileptics	627 (16.9)	23 661 (9.2)	4080 (21.2)	7134 (35.6)	35 502 (11.8)	1300 (35.1)	33 036 (12.9)	6672 (34.7)	8674 (43.3)	49 682 (16.6)
N05 psycholeptics	1914 (51.6)	98 049 (38.2)	11 065 (57.6)	14 782 (73.8)	125 810 (42.0)	3193 (86.1)	113 820 (44.3)	14 672 (76.4)	16 149 (80.7)	147 834 (49.3)
N06A antidepressants	1691 (45.6)	83 376 (32.5)	10 015 (52.1)	14 142 (70.6)	109 224 (36.4)	2338 (63.1)	98 832 (38.5)	12 860 (67.0)	15 452 (77.2)	129 482 (43.2)
N06B psychostimulants	<5	152 (0.1)	17 (0.9)	40 (0.2)	211 (0.1)	6 (0.2)	168 (0.1)	20 (0.1)	44 (0.2)	238 (0.1)

Like that observed for comorbidities, the use of drugs for chronic disease treatments, such as drugs for diabetes and beta blockers, was highest in the high-decreasing cluster at the last observation, followed by the intermediate-slow/increasing. The highest rise in patients’ drug use between baseline and end of follow-up was observed in the intermediate-increasing clusters.

### Model testing in Aurum and IPCI

#### Aurum

There were 265 101 patients included from Aurum. They had similar baseline characteristics to those in GOLD. However, the Aurum population had a higher CCI [mean 1.45 (SD 1.69)] compared to GOLD (Table 1).

Compared to GOLD, Aurum patients had a longer median follow-up of 5.0 years [IQR 3.9–5.0], with 16.4% dying during the follow-up period. The proportions of patients belonging to each of the resultant clusters in Aurum were similar to GOLD, with an increase of 1.4% in the intermediate-slow/increasing and a reduction of 0.8% in the high-decreasing Aurum clusters (Table 1).

Aurum participants showed good fit of the data; >50% had posterior probability ≥0.7 (Appendix 2; Table S5). The resultant clusters had similar polypharmacy trajectories and associated mortality risks to those in GOLD (Appendix 2; Figure S1 and Table S6).

Tables S7 and S8 in Appendix 2 describe comorbidities and drug burden in Aurum at baseline and at end of follow-up. Aurum clusters showed similar comorbidity and medication profiles to those in GOLD. For example, the low-steady cluster in Aurum had the lowest CCI at baseline and the slowest change over time [mean 1.3 (SD 1.5), Δ = 0.5]. The intermediate-fast/increasing, on the other hand, had the highest baseline cancer diagnosis and onset throughout the study period. Proportion of patients with any malignancy at baseline was 32.1%, jumping to 56.5% by the end of follow-up (Δ = 24.4%).

#### IPCI

There were 139 307 patients identified in IPCI. This Dutch population had similar characteristics to those from the UK, with lower median number of prescribed ingredients: 6 [IQR 3–10] (Table 1).

The median follow-up was 5.0 years [IQR 3.4–5.0], with 13.4% dying during follow-up. The distribution of patients in each cluster was slightly different. More patients belonged to the low-steady cluster compared to UK databases: 90.3% in IPCI vs. 85.7% and 85.3% in GOLD and Aurum. Additionally, a considerably lower proportion of patients belonged to the high-decreasing cluster: 2.7% in IPCI vs. 5%–6% in GOLD and Aurum databases (Table 1).

Like in Aurum, IPCI participants showed good fit of the data, similar polypharmacy trajectories and associated mortality risks (Appendix 2; Table S5; Figure S2 and Table S6).

Appendix 2; Tables S9 and S10 described comorbidity and drug burden at baseline and end of follow-up for all IPCI patients and by individual cluster. Overall, the IPCI study population had some differences in disease prevalence compared to those from the UK databases. They had a lower recorded prevalence of depression, renal and chronic kidney diseases, and asthma, but a higher prevalence of pneumonia. IPCI population also had different prevalence and accumulation rates of drug use, including opioids, psycholeptics, anti-thrombotics, diuretics, anti-bacterial and anti-inflammatory drugs.

The individual IPCI clusters exhibited similar comorbidity and drug prescription patterns to their counterparts in GOLD and Aurum. For example, the low-steady cluster had the lowest CCI at baseline and the slowest increase over the study period, whilst the intermediate-fast/increasing cluster had the highest prevalence and risk of cancer diagnosis over the study period.

## Discussion

### Principal findings

This study identified four clusters of older people with distinct polypharmacy trajectories and associated mortality risk. The emerging clusters were low-steady, intermediate-slow/increasing, intermediate-fast/increasing and high-decreasing. The clusters were validated in two independent databases and had similar polypharmacy trajectories, mortality risks, and clinical characteristics.

#### Clusters characteristics

The low-steady cluster included the highest proportion of the study population. Similar findings were observed in previous research [14]. Compared to the low-steady cluster, the other groups—by definition—had higher use of different drugs. Using some of these drugs, such as opioids, antidepressants and psycholeptics is associated with higher mortality in older people with polypharmacy [29]. Patients in the other clusters had higher burden of cognitive impairment (e.g. dementia) and mental problems, which are associated with chronic diseases and polypharmacy [1, 15, 30].

The intermediate-fast/increasing cluster had the highest prevalence and incidence of cancer (>50%) and highest mortality, with > 80% dying during follow-up. Patients with cancer have a higher risk of polypharmacy and potentially inappropriate medications [31–33]. A study monitoring the accumulation of polypharmacy in the year prior to death found that older people who died from cancer had the highest increase in polypharmacy [13].

The intermediate-slow/increasing cluster had the most rapid accumulation of non-cancer chronic diseases. In general, new diagnosis of a chronic disease is accompanied by an increase in polypharmacy [34]. A study found that mean prescribed medications increased from 5.0 to 6.6 after a diabetes diagnosis. The increase was mainly due to the use of diabetes treatment, in addition to anti-hypertensives and lipid-lowering agents [34].

Shortly after a new chronic disease diagnosis, multiple lines of therapy might be tested before finding the optimal treatment [35, 36]. Moreover, physicians tend to deprescribe for older patients with multimorbidity [37]. This could explain the high baseline value and decreasing polypharmacy trend in the high-decreasing cluster, as this cluster has the highest baseline prevalence of chronic diseases.

The high-decreasing and intermediate-slow/increasing clusters had overlapping HRs (≥4.0). Previous studies produced supporting evidence on the association between older patients with chronic diseases and polypharmacy, and the increased risk of death [38].

#### External validation of the models in Aurum and IPCI

Good validation was observed in Aurum and IPCI. The identified clusters were replicated and had similar mortality risks.

In IPCI, the proportions of patients in each cluster were slightly different, with a higher proportion belonging to the low-steady cluster; 90% vs. ~85% in UK data. This led to lower proportions of patients in the other more morbid clusters. Unlike the UK population, anxiety and dementia burden in the low-steady cluster were comparable to the other clusters.

The biggest differences in disease prevalence were exhibited in renal and chronic kidney diseases, lower in IPCI, and pneumonia, higher in IPCI. Use of most drugs was lower in IPCI. For example, compared to GOLD and Aurum, patients in the intermediate-increasing clusters had a much lower prevalence of nervous system drugs like opioids at baseline but a higher increment over the study period.

Although the individual clusters from the Dutch and UK data had different disease burden and drug use prevalence, they exhibited similar accumulation patterns of comorbidity burden and drug use over the study period. The difference between the UK and Dutch data can be attributed to different populations, healthcare guidelines and data entry systems and practices.

### Strengths, limitations and implications

A strength of the study is using JLCM, which performed well with good discrimination and generalisable clusters. Traditional longitudinal models fail to simultaneously account for censored data and heterogeneity of the older population. The good external validation in Aurum and IPCI supports the generalisability of the clusters. Despite the differences in study populations and health practices, the resulting clusters had similar characteristics and polypharmacy trends. Using a common data model facilitated the model validation.

Using UK and Dutch primary care databases has strengths and limitations. In both countries, general practitioners (GPs) are the first point of contact for patients, and routinely collected comprehensive information is difficult to find elsewhere. GOLD, Aurum and IPCI are representative of their respective general population. Consequently, they are frequently used in longitudinal studies, providing good-quality records for research [18, 19, 39].

Using primary care data for calculating polypharmacy means a loss of information on over-the-counter drugs and, potentially, secondary care information. Additionally, the primary care databases used in this study provide information on prescriptions, not dispensations. These limitations might have led to an underestimated polypharmacy count.

An implication of this study is that monitoring polypharmacy over time can accurately identify the vulnerable and deteriorating subgroups within the older population. Polypharmacy was estimated by calculating the total ingredients prescribed annually. This approach might overestimate the actual number of ingredients a patient is taking at any point in time. Ageing and chronic conditions can affect the pharmacokinetics and pharmacodynamics of treatment [40, 41]. Therefore, it is important to account for the cumulative effect of polypharmacy on an old, vulnerable population [41].

Whilst a higher mortality risk cannot be solely attributed to the accumulation of drug intake in a patient, polypharmacy can be used as a proxy to identify vulnerable patients with higher comorbidities or frailty burden. This was observed in the high-decreasing cluster, which, despite its decreasing polypharmacy trend, had a high HR compared to the low-steady cluster. Trajectory of polypharmacy is also a sign of incident multimorbidity as observed in the characteristics of both the intermediate-increasing clusters and supported by Vos *et al*. [42]. The overlap between polypharmacy and frailty have been discussed extensively [43–46]. We used polypharmacy as a single measure to address these phenomena. This allowed us to identify clusters with unique characteristics as several ingredients may be used to treat the same condition, giving more weight to that condition. This cannot be seen with multimorbidity or frailty scores, as they usually address the general physical functionality caused by the accumulation of multiple comorbidities/deficits rather than specific conditions. However, in our results, we described multimorbidity burden (CCI), and clinical conditions, most of which are considered in frailty indices [47].

Guidelines and interventions are available for optimising polypharmacy in older people with cancer and/or chronic diseases [31, 48]. Optimising prescriptions by identifying inappropriate and redundant prescriptions can improve the general condition, health status and functional level in the targeted patients [49]. The earlier these interventions are targeted, the better the results [45]. Our findings can aid in the early identification of target populations and understanding their prognosis. This will help physicians tailor more appropriate treatment packages and optimise the use of limited resources.

## Conclusion

This study showed that clustering older people based on their polypharmacy progression over time provides a useful tool to identify those most vulnerable and at higher risk of mortality. Polypharmacy is an indicator for multimorbidity and a predictor for potential adverse events.

## Supplementary Material

aa_25_0397_File002_afaf233

aa_25_0397_File003_afaf233

## Data Availability

Data were obtained from CPRD under the Oxford University CPRD licence. Direct data sharing is not allowed. Data access can be obtained from CPRD, conditional on eRAP approval. IPCI data were accessed through a collaboration with Erasmus MC. Direct data sharing is not allowed.
