# Supplementary material for: Longitudinal trajectories of polypharmacy in older people, and their association with the risk of mortality: a joint latent class model analysis of real-world data from the UK and the Netherlands
Source: Age Ageing. 2025 Aug 20;54(8):afaf233. doi: 10.1093/ageing/afaf233 (PMC12365978; doi:10.1093/ageing/afaf233)
Supplement: aa_25_0397_File002_afaf233 [file aa_25_0397_file002_afaf233.docx]

**Longitudinal trajectories of polypharmacy in older people, and their association with the risk of mortality: A joint latent class model analysis of real-world data from the UK and the Netherlands**

**Appendix 1**

**Clinical Practice Research Datalink (CPRD)**

CPRD is a data provider that collects fully coded anonymised EHR from UK primary care practices. These data are representative of the UK population demographics (i.e., sex, age and ethnicity).

There are two observational primary care databases contributing to CPRD: GOLD and Aurum [1, 2]. CPRD GOLD contains data from General practitioners (GP) practices that use the Vision® electronic health record (EHR) software while Aurum’s data come from GP practices using EMIS Web® EHR software. Aurum’s coverage includes practices from England only. When it was launched in 2019, Aurum contained data from >19 million of the current and historical English population (patients acceptable for clinical research), with 7 million active patients representing 13% of the English population. CPRD GOLD -previously known as VAMP, GPRD and CPRD- was launched in 1987. By September 2019, it contained data from 17.5 million acceptable patients from all four UK nations, with 2.8 million active patients representing 4.3% of the UK population [3]. However, this coverage has changed dramatically in recent years, with many GP practices migrating their data to Aurum, leaving GOLD to cover only seven English practices in January 2024. As a result of this migration, some practices have contributed data to both databases, leading to a small proportion of overlapping practices, whose CPRD identifiers are disclosed by CPRD to researchers using both databases for their studies [[link](https://www.cprd.com/sites/default/files/2022-02/CPRD%20Aurum%20FAQs%20v2.2.pdf)].

**The Integrated Primary Care Information (IPCI)**

IPCI is a Dutch primary care database. It contains anonymised longitudinal data from General practitioner (GP) records. IPCI data contains information on patient demographics, symptoms and diagnoses, laboratory tests, prescriptions, and correspondences with secondary care. IPCI was started in 1992 by the Department of Medical Informatics of the Erasmus University Medical Centre in Rotterdam. The currently available IPCI data come from 2006 onwards, as the number of practices joining IPCI in its first decade was limited. As of July 2021, IPCI contains 2.5 million patient records with 1.4 million active patients, covering 8.1% of the Dutch population. IPCI is representative of the Dutch population, with its practices covering mostly the central parts of the country, but it also has good coverage of non-urban areas [4].

**Methods for identifying polypharmacy**

Table S1 Methods applied to identify polypharmacy in Source CPRD GOLD and OMOP-CDM mapping

|  | Source | OMOP-CDM |
| --- | --- | --- |
| Drug level | Substance | Ingredient |
| Example (paracetamol + aspirin) | One substance | Two different ingredients |
| Example (paracetamol, aspirin, paracetamol + aspirin) | Three different substances | Two different ingredients |
| Doses | Different doses count the same | Different doses count the same |

**References**

1. Herrett E, Gallagher AM, Bhaskaran K, Forbes H, Mathur R, van Staa T, Smeeth L: **Data Resource Profile: Clinical Practice Research Datalink (CPRD)**. *International Journal of Epidemiology* 2015, **44**(3):827-836.

2. Wolf A, Dedman D, Campbell J, Booth H, Lunn D, Chapman J, Myles P: **Data resource profile: Clinical Practice Research Datalink (CPRD) Aurum**. *International Journal of Epidemiology* 2019, **48**(6):1740-1740g.

3. **Release Notes: CPRD GOLD September 2019** [<https://cprdcw.cprd.com/_docs/Release_Notes_September2019.pdf>]

4. de Ridder MAJ, de Wilde M, de Ben C, Leyba AR, Mosseveld BMT, Verhamme KMC, van der Lei J, Rijnbeek PR: **Data Resource Profile: The Integrated Primary Care Information (IPCI) database, The Netherlands**. *International Journal of Epidemiology* 2022, **51**(6):e314-e323.
