# Supplementary material for: Longitudinal trajectories of polypharmacy in older people, and their association with the risk of mortality: a joint latent class model analysis of real-world data from the UK and the Netherlands
Source: Age Ageing. 2025 Aug 20;54(8):afaf233. doi: 10.1093/ageing/afaf233 (PMC12365978; doi:10.1093/ageing/afaf233)
Supplement: aa_25_0397_File003_afaf233 [file aa_25_0397_file003_afaf233.docx]

**Longitudinal trajectories of polypharmacy in older people, and their association with the risk of mortality: A joint latent class model analysis of real-world data from the UK and the Netherlands**

**Appendix 2**

Table S2 Comparison between different cluster numbers for joint latent class models in GOLD

| Number of clusters | BIC | Entropy | Smallest cluster size (%) | More than 50% of the population has a posterior probability >0.7 |
| --- | --- | --- | --- | --- |
| 1 | 8446892 | 1.00 | NA | NA |
| 2 | 8399786 | 0.84 | 10.0 | Yes |
| 3 | 8368178 | 0.84 | 4.8 | Yes |
| 4 | 8358633 | 0.82 | 1.2 | Yes |
| 5* | NA | NA | NA | NA |
| * Did not converge | | | | |

Table S3 Joint latent class model results in GOLD (including baseline age and gender)

| N eligible=299859 | N events=38031 | N observations=1350561 |
| --- | --- | --- |
| Longitudinal model | Parameter | Estimate (95% CI) |
|  | Intermediate-fast/increasing | Intercept = 10.70 (10.55, 10.85) |
|  |  | Slope = 6.40 (6.33, 6.47) |
|  | Low-steady | Intercept = 5.97 (5.95, 6.00) |
|  |  | Slope = 0.07 (0.06, 0.07) |
|  | Intermediate-slow/increasing | Intercept = 11.22 (11.11, 11.32) |
|  |  | Slope = 1.98 (1.95, 2.00) |
|  | High-decreasing | Intercept = 23.44 (23.35, 23.54) |
|  |  | Slope = -1.75 (-1.77, -1.73) |
|  | Gender=male | -0.51 (-0.53, -0.49) |
|  | Age-65* | 0.16 (0.16, 0.16) |
| Survival model | Parameter | HR (95% CI) |
|  | Low-steady | Ref |
|  | Intermediate-fast/increasing | 20.53 (19.87, 21.21) |
|  | Intermediate-slow/increasing | 4.95 (4.84, 5.07) |
|  | High-decreasing | 4.64 (4.58, 4.73) |
|  | Gender=male | 1.36 (1.34, 1.38) |
|  | Age-65* | 1.17 (1.17, 1.17) |

* Continuous, centred around 65

Table S4 Summary polypharmacy and number of deaths during study follow-up for all clusters in GOLD

|  | | Intermediate-fast/increasing  N=3708 | Low-steady N=256923 | Intermediate-slow/increasing  N=19207 | High-decreasing N=20021 | Overall N=299859 |
| --- | --- | --- | --- | --- | --- | --- |
| Follow-up time (median (IQR)) | | 1.7 (0.3, 2.8) | 4.3 (1.4, 5.0) | 4.6 (2.4, 5.0) | 2.5 (1.0, 5.0) | 4.1 (1.4, 5.0) |
| Death (n (%)) | Year 1 | 1049 (28.3) | 5274 (2.1) | 2005 (10.4) | 2224 (11.1) | 10552 (3.5) |
|  | Year 2 | 2108 (56.9) | 9497 (3.7) | 3492 (18.2) | 3748 (18.7) | 18845 (6.3) |
|  | Year 3 | 2718 (73.3) | 13130 (5.1) | 5188 (27.0) | 4900 (24.5) | 25936 (8.6) |
|  | Year 4 | 2988 (80.6) | 16496 (6.4) | 6938 (36.1) | 5882 (29.4) | 32304 (10.8) |
|  | Year 5 | 3107 (83.8) | 19648 (7.6) | 8612 (44.8) | 6664 (33.3) | 38031 (12.7) |
| Polypharmacy (median (IQR)) | Year 1 | 20 (12, 26) | 7 (3, 11) | 16 (11, 21) | 24 (20, 29) | 8 (4, 13) |
|  | Year 2 | 24 (17, 30) | 7 (3, 11) | 18 (13, 23) | 22 (17, 27) | 8 (4, 13) |
|  | Year 3 | 28 (21, 35) | 7 (3, 11) | 20 15, 25) | 20 (15, 25) | 8 (4, 13) |
|  | Year 4 | 33 (27, 40) | 7 (3, 11) | 22 (18, 27) | 18 (13, 24) | 8 (4, 14) |
|  | Year 5 | 37 (30, 44) | 7 (3, 11) | 23 (19, 28) | 17 (12, 23) | 8 (4, 14) |

Table S5 Posterior probability in each cluster in Aurum and IPCI

| n (%) | Intermediate-fast/increasing | Low-steady | Intermediate-slow/increasing | High-decreasing |
| --- | --- | --- | --- | --- |
| CPRD Aurum | | | | |
| Posterior probability ≥ 0.7 | 1919 (56.5) | 210692 (93.5) | 11498 (55.7) | 11041 (70.4) |
| IPCI | | | | |
| Posterior probability ≥ 0.7 | 1130 (79.1) | 122519 (97.4) | 4778 (57.8) | 2917 (77.3) |

Table S6 Summary polypharmacy and number of deaths during study follow-up for all clusters in Aurum and IPCI

| Aurum | | | | | | |
| --- | --- | --- | --- | --- | --- | --- |
|  | | Intermediate-fast/increasing  N=3396 | Low-steady N=225383 | Intermediate-slow/increasing N=20639 | High-decreasing N=15683 | Overall N=265101 |
| Follow-up time (median (IQR)) | | 1.7 (1.0, 2.6) | 5.0 (4.5, 5.0) | 4.8 (2.7, 5.0) | 4.1 (1.8, 5.0) | 5.0 (3.9, 5.0) |
| Death (n (%)) | Year 1 | 974 (28.7) | 5212 (2.3) | 1923 (9.3) | 1881 (12.0) | 9990 (3.8) |
|  | Year 2 | 2127 (62.6) | 9947 (4.4) | 3585 (17.4) | 3342 (21.3) | 19001 (7.2) |
|  | Year 3 | 2733 (80.5) | 14662 (6.5) | 5605 (27.2) | 4617 (29.4) | 27617 (10.4) |
|  | Year 4 | 2966 (87.3) | 19342 (8.6) | 7848 (38.0) | 5716 (36.4) | 35872 (13.5) |
|  | Year 5 | 3052 (89.9) | 23924 (10.6) | 9832 (47.6) | 6538 (41.7) | 43346 (16.4) |
| Polypharmacy (median (IQR)) | Year 1 | 19 (13, 26) | 7 (3, 11) | 15 (11, 20) | 24 (20, 29) | 8 (4, 13) |
|  | Year 2 | 24 (17, 30) | 7 (3, 11) | 17 (12, 22) | 22 (18, 28) | 8 (4, 13) |
|  | Year 3 | 29 (22, 35) | 7 (3, 11) | 20 (15, 25) | 20 (15, 26) | 8 (4, 13) |
|  | Year 4 | 32 (26, 39) | 7 (3, 11) | 22 (17, 26) | 18 (14, 24) | 8 (4, 13) |
|  | Year 5 | 37 (32, 45) | 7 (3, 11) | 23 (19, 28) | 17 (12, 22) | 8 (4, 13) |
| IPCI | | | | | | |
|  | | Intermediate-fast/increasing  N=1429 | Low-steady N=125835 | Intermediate-slow/increasing N=8268 | High-decreasing N=3775 | Overall N=139307 |
| Follow-up time (median (IQR)) | | 1.6 (0.9, 2.5) | 5.0 (3.8, 5.0) | 5.0 (3.0, 5.0) | 3.8 (1.7, 5.0) | 5.0 (3.4, 5.0) |
| Death (n (%)) | Year 1 | 455 (31.8) | 2731 (2.2) | 605 (7.3) | 396 (10.5) | 4187 (3.0) |
|  | Year 2 | 936 (65.5) | 5307 (4.2) | 1202 (14.5) | 754 (20.0) | 8199 (5.9) |
|  | Year 3 | 1171 (81.9) | 7765 (6.2) | 1970 (23.8) | 1014 (26.9) | 11920 (8.6) |
|  | Year 4 | 1263 (88.4) | 10072 (8.0) | 2831 (34.2) | 1277 (33.8) | 15443 (11.1) |
|  | Year 5 | 1288 (90.1) | 12304 (9.8) | 3633 (43.9) | 1493 (39.5) | 18718 (13.4) |
| Polypharmacy (median (IQR)) | Year 1 | 17 (10, 24) | 6 (3, 10) | 13 (8, 18) | 23 (19, 27) | 6 (3, 11) |
|  | Year 2 | 22 (16, 27) | 6 (3, 10) | 15 (10, 20) | 20 (16, 25) | 6 (3, 11) |
|  | Year 3 | 26 (21, 31) | 6 (3, 10) | 18 (13, 22) | 18 (14, 23) | 7 (3, 11) |
|  | Year 4 | 30 (24, 35) | 6 (3, 10) | 20 (16, 24) | 17 (12, 21) | 7 (3, 11) |
|  | Year 5 | 33 (29, 39) | 6 (3, 10) | 22 (18, 26) | 16 (11, 20) | 7 (3, 11) |

Figure S1 Intercepts and slopes of ingredients, and (b) Survival probabilities of each cluster in Aurum


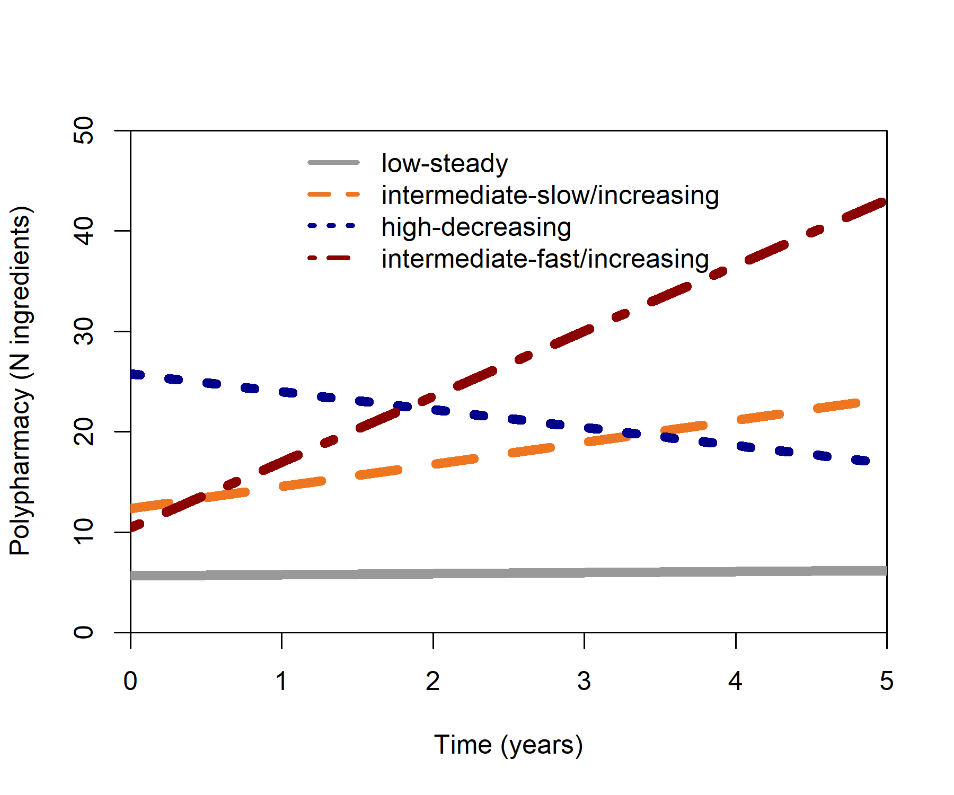


(a)


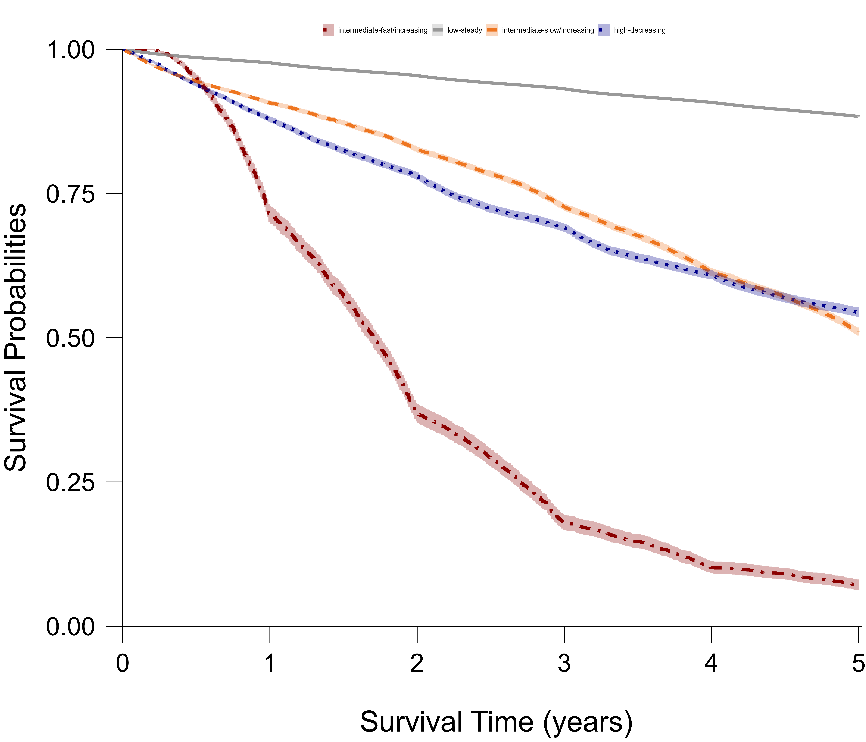


(b)

Table S7 Individual comorbidities at baseline and the last observation in Aurum

|  | At baseline | | | | | At the last observation | | | | |
| --- | --- | --- | --- | --- | --- | --- | --- | --- | --- | --- |
|  | Medium-fast/increasing  N=3396 | Low-steady N=225383 | Medium-slow/increasing N=20639 | High-decreasing N=15683 | Overall N=265101 | Medium-fast/increasing  N=3396 | Low-steady N=225383 | Medium-slow/increasing N=20639 | High-decreasing N=15683 | Overall N=265101 |
| Mean (SD) | | | | | | | | | | |
| Charlson morbidity index | 2.48 (2.10) | 1.26 (1.54) | 2.31 (1.96) | 2.83 (2.10) | 1.45 (1.69) | 3.98 (2.67) | 1.78 (1.86) | 3.56 (2.41) | 3.55 (2.33) | 2.05 (2.05) |
| n (%) | | | | | | | | | | |
| Myocardial infarction | 339 (10.0) | 11027 (4.9) | 2117 (10.3) | 2177 (13.9) | 15660 (5.9) | 448 (13.2) | 13866 (6.2) | 2990 (14.5) | 2544 (16.2) | 19848 (7.5) |
| Congestive heart failure | 289 (8.5) | 5843 (2.6) | 1550 (7.5) | 1931 (12.3) | 9613 (3.6) | 537 (15.8) | 11346 (5.0) | 3522 (17.1) | 2840 (18.1) | 18245 (6.9) |
| Peripheral vascular disease | 180 (5.3) | 3917 (1.7) | 971 (4.7) | 939 (6.0) | 6007 (2.3) | 231 (6.8) | 5596 (2.5) | 1435 (7.0) | 1194 (7.6) | 8456 (3.2) |
| Cerebrovascular disease | 476 (14.0) | 15774 (7.0) | 2938 (14.2) | 2785 (17.8) | 21973 (8.3) | 619 (18.2) | 22832 (10.1) | 4237 (20.5) | 3433 (21.9) | 31121 (11.7) |
| Dementia | 238 (7.0) | 7392 (3.3) | 1303 (6.3) | 1217 (7.8) | 10150 (3.8) | 399 (11.7) | 16084 (7.1) | 2705 (13.1) | 2243 (14.3) | 21431 (8.1) |
| Chronic obstructive pulmonary disease | 938 (27.6) | 32567 (14.4) | 6047 (29.3) | 5697 (36.3) | 45249 (17.1) | 1094 (32.2) | 40644 (18.0) | 7706 (37.3) | 6362 (40.6) | 55806 (21.1) |
| Rheumatologic disease | 210 (6.2) | 8882 (3.9) | 1536 (7.4) | 1556 (9.9) | 12184 (4.6) | 242 (7.1) | 11688 (5.2) | 2082 (10.1) | 1763 (11.2) | 15775 (6.0) |
| Peptic ulcer disease | 272 (8.0) | 10874 (4.8) | 1670 (8.1) | 1531 (9.8) | 14347 (5.4) | 333 (9.8) | 12455 (5.5) | 2024 (9.8) | 1713 (10.9) | 16525 (6.2) |
| Mild liver disease | 37 (1.1) | 729 (0.3) | 179 (0.9) | 162 (1.0) | 1107 (0.4) | 65 (1.9) | 1147 (0.5) | 310 (1.5) | 233 (1.5) | 1755 (0.7) |
| Diabetes with chronic complications | 495 (14.6) | 14324 (6.4) | 3306 (16.0) | 3487 (22.2) | 21612 (8.2) | 601 (17.7) | 19206 (8.5) | 4418 (21.4) | 4180 (26.7) | 28405 (10.7) |
| Hemiplegia or paraplegia | 18 (0.5) | 439 (0.2) | 93 (0.5) | 104 (0.7) | 654 (0.2) | 22 (0.6) | 493 (0.2) | 112 (0.5) | 110 (0.7) | 737 (0.3) |
| Renal disease | 897 (26.4) | 38476 (17.1) | 5909 (28.6) | 5441 (34.7) | 50723 (19.1) | 1156 (34.0) | 53581 (23.8) | 8329 (40.4) | 6843 (43.6) | 69909 (26.4) |
| Any malignancy | 1089 (32.1) | 38666 (17.2) | 4873 (23.6) | 3712 (23.7) | 48340 (18.2) | 1919 (56.5) | 54626 (24.2) | 7880 (38.2) | 4688 (29.9) | 69113 (26.1) |
| Moderate to severe liver disease | 16 (0.5) | 248 (0.1) | 63 (0.3) | 63 (0.4) | 390 (0.1) | 31 (0.9) | 466 (0.2) | 130 (0.6) | 96 (0.6) | 723 (0.3) |
| Metastatic solid tumour | 67 (2.0) | 397 (0.2) | 121 (0.6) | 108 (0.7) | 693 (0.3) | 343 (10.1) | 1442 (0.6) | 745 (3.6) | 229 (1.5) | 2759 (1.0) |
| AIDS | 0 (0) | 20 (0) | 5 (0) | <5 | 28 (0) | 0 (0) | 20 (0) | 6 (0) | <5 | 30 (0) |
| Hypertension | 1766 (52) | 102870 (45.6) | 11677 (56.6) | 9586 (61.1) | 125899 (47.5) | 1866 (54.9) | 117760 (52.2) | 12946 (62.7) | 10201 (65) | 142773 (53.9) |
| Heart failure | 286 (8.4) | 6011 (2.7) | 1563 (7.6) | 1942 (12.4) | 9802 (3.7) | 547 (16.1) | 11922 (5.3) | 3625 (17.6) | 2897 (18.5) | 18991 (7.2) |
| Osteoporosis | 280 (8.2) | 13115 (5.8) | 1967 (9.5) | 2193 (14.0) | 17555 (6.6) | 385 (11.3) | 19805 (8.8) | 3329 (16.1) | 2792 (17.8) | 26311 (9.9) |
| Gastroesophageal reflux disease | 165 (4.9) | 8678 (3.9) | 1195 (5.8) | 1381 (8.8) | 11419 (4.3) | 212 (6.2) | 11650 (5.2) | 1648 (8.0) | 1705 (10.9) | 15215 (5.7) |
| Chronic kidney disease | 836 (24.6) | 36490 (16.2) | 5558 (26.9) | 5027 (32.1) | 47911 (18.1) | 991 (29.2) | 49708 (22.1) | 7423 (36.0) | 6132 (39.1) | 64254 (24.2) |
| Venous thromboembolism | 235 (6.9) | 8891 (3.9) | 1373 (6.7) | 1483 (9.5) | 11982 (4.5) | 482 (14.2) | 12818 (5.7) | 2401 (11.6) | 1939 (12.4) | 17640 (6.7) |
| Hypothyroidism | 339 (10) | 17814 (7.9) | 2300 (11.1) | 2166 (13.8) | 22619 (8.5) | 390 (11.5) | 21335 (9.5) | 2819 (13.7) | 2433 (15.5) | 26977 (10.2) |
| Stroke | 221 (6.5) | 6631 (2.9) | 1278 (6.2) | 1254 (8.0) | 9384 (3.5) | 337 (9.9) | 10828 (4.8) | 2183 (10.6) | 1692 (10.8) | 15040 (5.7) |
| Anxiety | 480 (14.1) | 26971 (12.0) | 3646 (17.7) | 3543 (22.6) | 34640 (13.1) | 604 (17.8) | 32831 (14.6) | 4674 (22.6) | 4140 (26.4) | 42249 (15.9) |
| Asthma | 436 (12.8) | 20086 (8.9) | 3638 (17.6) | 3798 (24.2) | 27958 (10.5) | 466 (13.7) | 22306 (9.9) | 4070 (19.7) | 4038 (25.7) | 30880 (11.6) |
| Pneumonia | 225 (6.6) | 5282 (2.3) | 1202 (5.8) | 1401 (8.9) | 8110 (3.1) | 594 (17.5) | 11664 (5.2) | 3498 (16.9) | 2714 (17.3) | 18470 (7) |
| Diabetes | 791 (23.3) | 29705 (13.2) | 5406 (26.2) | 5246 (33.5) | 41148 (15.5) | 918 (27.0) | 37519 (16.6) | 6555 (31.8) | 5856 (37.3) | 50848 (19.2) |
| Inflammatory bowel disease | 38 (1.1) | 1953 (0.9) | 304 (1.5) | 256 (1.6) | 2551 (1.0) | 41 (1.2) | 2274 (1.0) | 366 (1.8) | 295 (1.9) | 2976 (1.1) |
| Depressive disorder | 467 (13.8) | 22493 (10.0) | 3362 (16.3) | 3427 (21.9) | 29749 (11.2) | 548 (16.1) | 25902 (11.5) | 4129 (20) | 3894 (24.8) | 34473 (13.0) |

Table S8 Drug use by drug classes and common drugs at baseline and the last observation for each cluster and the overall population in Aurum

|  | At baseline (n (%)) | | | | | | At the last observation (n (%)) | | | | | |  |
| --- | --- | --- | --- | --- | --- | --- | --- | --- | --- | --- | --- | --- | --- |
|  | Medium-fast/increasing  N=3396 | Low-steady N=225383 | Medium-slow/increasing N=20639 | High-decreasing N=15683 | Overall N=265101 | Medium-fast/increasing  N=3396 | | Low-steady N=225383 | Medium-slow/increasing N=20639 | High-decreasing N=15683 | Overall N=265101 |  |  |
| Drug classes (ATC name) | | | | | | | | | | | | | |
| A: Alimentary tract and metabolism | 2691 (79.2) | 133883 (59.4) | 17729 (85.9) | 15639 (99.7) | 169942 (64.1) | 3387 (99.7) | | 141200 (62.6) | 20446 (99.1) | 14839 (94.6) | 179872 (67.9) |  |  |
| B: Blood and blood forming organs | 1846 (54.4) | 72501 (32.2) | 11875 (57.5) | 11817 (75.3) | 98039 (37.0) | 2501 (73.6) | | 85037 (37.7) | 15355 (74.4) | 11102 (70.8) | 113995 (43.0) |  |  |
| C: Cardiovascular system | 2653 (78.1) | 148365 (65.8) | 17496 (84.8) | 14762 (94.1) | 183276 (69.1) | 2906 (85.6) | | 158402 (70.3) | 18545 (89.9) | 13807 (88.0) | 193660 (73.1) |  |  |
| D: Dermatological | 1391 (41.0) | 67817 (30.1) | 10332 (50.1) | 12346 (78.7) | 91886 (34.7) | 2064 (60.8) | | 61692 (27.4) | 13056 (63.3) | 9003 (57.4) | 85815 (32.4) |  |  |
| G: Genito-urinary system and sex hormones | 654 (19.3) | 36038 (16.0) | 4927 (23.9) | 5655 (36.1) | 47274 (17.8) | 817 (24.1) | | 39008 (17.3) | 6189 (30.0) | 4401 (28.1) | 50415 (19.0) |  |  |
| H: Systemic hormonal preparations, excluding sex hormones and insulins | 478 (14.1) | 24534 (10.9) | 3400 (16.5) | 3552 (22.6) | 31964 (12.1) | 590 (17.4) | | 27414 (12.2) | 4234 (20.5) | 3302 (21.1) | 35540 (13.4) |  |  |
| J: Anti-infective for systemic use | 1836 (54.1) | 86716 (38.5) | 12512 (60.6) | 13615 (86.8) | 114679 (43.3) | 2774 (81.7) | | 78796 (35.0) | 16023 (77.6) | 10193 (65.0) | 107786 (40.7) |  |  |
| L: Antineoplastic and immunomodulation agents | 310 (9.1) | 8762 (3.9) | 1451 (7.0) | 1456 (9.3) | 11979 (4.5) | 400 (11.8) | | 11193 (5.0) | 2180 (10.6) | 1195 (7.6) | 14968 (5.6) |  |  |
| M: Musculoskeletal system | 1162 (34.2) | 64731 (28.7) | 8840 (42.8) | 9733 (62.1) | 84466 (31.9) | 1557 (45.8) | | 58211 (25.8) | 10218 (49.5) | 7184 (45.8) | 77170 (29.1) |  |  |
| N: Nervous system | 2202 (64.8) | 99736 (44.3) | 15178 (73.5) | 14615 (93.2) | 131731 (49.7) | 3298 (97.1) | | 100508 (44.6) | 18680 (90.5) | 13433 (85.7) | 135919 (51.3) |  |  |
| P: Anti-parasitic products, insecticides and repellents | 310 (9.1) | 8762 (3.9) | 1451 (7.0) | 1456 (9.3) | 11979 (4.5) | 400 (11.8) | | 11193 (5.0) | 2180 (10.6) | 1195 (7.6) | 14968 (5.6) |  |  |
| R: Respiratory system | 1644 (48.4) | 70774 (31.4) | 11792 (57.1) | 12438 (79.3) | 96648 (36.5) | 2741 (80.7) | | 68829 (30.5) | 15175 (73.5) | 10261 (65.4) | 97006 (36.6) |  |  |
| S: Sensory organs | 451 (13.3) | 22257 (9.9) | 3347 (16.2) | 4294 (27.4) | 30349 (11.4) | 1244 (36.6) | | 24932 (11.1) | 5248 (25.4) | 3351 (21.4) | 34775 (13.1) |  |  |
| V: Various | 7 (0.2) | 123 (0.1) | 38 (0.2) | 104 (0.7) | 272 (0.1) | 18 (0.5) | | 125 (0.1) | 77 (0.4) | 86 (0.5) | 306 (0.1) |  |  |
| Drugs (ATC name) | | | | | | | | | | | | | |
| A02 Drugs for acid related disorder | 2390 (70.4) | 125830 (55.8) | 15750 (76.3) | 14226 (90.7) | 158196 (59.7) | 3048 (89.8) | | 149789 (66.5) | 18564 (89.9) | 14718 (93.8) | 186119 (70.2) |  |  |
| A10A/ A10B Drugs used in diabetes | 701 (20.6) | 24224 (10.7) | 4779 (23.2) | 4889 (31.2) | 34593 (13.0) | 828 (24.4) | | 29754 (13.2) | 5711 (27.7) | 5309 (33.9) | 41602 (15.7) |  |  |
| B01A Anti-thrombotic agents | 1167 (34.4) | 43490 (19.3) | 7327 (35.5) | 7215 (46.0) | 59199 (22.3) | 1801 (53.0) | | 66111 (29.3) | 11620 (56.3) | 8844 (56.4) | 88376 (33.3) |  |  |
| C03 Diuretics | 1805 (53.2) | 86546 (38.4) | 12381 (60.0) | 11340 (72.3) | 112072 (42.3) | 2295 (67.6) | | 99153 (44.0) | 15057 (73.0) | 12165 (77.6) | 128670 (48.5) |  |  |
| C07 Beta blocking agents | 1502 (44.2) | 75609 (33.5) | 9718 (47.1) | 8573 (54.7) | 95402 (36.0) | 1793 (52.8) | | 88270 (39.2) | 12096 (58.6) | 9479 (60.4) | 111638 (42.1) |  |  |
| C08 Calcium channel blockers | 1583 (46.6) | 81255 (36.1) | 10646 (51.6) | 9297 (59.3) | 102781 (38.8) | 1758 (51.8) | | 99561 (44.2) | 12468 (60.4) | 10043 (64.0) | 123830 (46.7) |  |  |
| C09 Agents renin-angiotensin systemic | 1868 (55.0) | 96993 (43.0) | 12616 (61.1) | 11139 (71.0) | 122616 (46.3) | 2045 (60.2) | | 110243 (48.9) | 14183 (68.7) | 11686 (74.5) | 138157 (52.1) |  |  |
| C10 Lipid modifying agents | 2144 (63.1) | 112230 (49.8) | 13983 (67.8) | 12128 (77.3) | 140485 (53.0) | 2312 (68.1) | | 133286 (59.1) | 15753 (76.3) | 12672 (80.8) | 164023 (61.9) |  |  |
| G03A Hormonal contraceptives for systemic use | 119 (3.5) | 11846 (5.3) | 1004 (4.9) | 853 (5.4) | 13822 (5.2) | 134 (3.9) | | 11999 (5.3) | 1034 (5.0) | 870 (5.5) | 14037 (5.3) |  |  |
| J01 Antibacterial systemic | 3143 (92.6) | 190797 (84.7) | 19526 (94.6) | 15454 (98.5) | 228920 (86.4) | 3328 (98.0) | | 203148 (90.1) | 20465 (99.2) | 15610 (99.5) | 242551 (91.5) |  |  |
| L01 Antineoplastic agents | 281 (8.3) | 11778 (5.2) | 2069 (10.0) | 2254 (14.4) | 16382 (6.2) | 326 (9.6) | | 13395 (5.9) | 2492 (12.1) | 2402 (15.3) | 18615 (7.0) |  |  |
| L04A Immunosuppressant | 117 (3.4) | 3953 (1.8) | 808 (3.9) | 926 (5.9) | 5804 (2.2) | 147 (4.3) | | 5181 (2.3) | 1173 (5.7) | 1083 (6.9) | 7584 (2.9) |  |  |
| M01A/ H02 Anti-inflammatory and/or anti-rheumatic | 2538 (74.7) | 154630 (68.6) | 16604 (80.4) | 13595 (86.7) | 187367 (70.7) | 2947 (86.8) | | 166706 (74) | 18110 (87.7) | 14084 (89.8) | 201847 (76.1) |  |  |
| N02A Opioids | 2451 (72.2) | 128988 (57.2) | 16289 (78.9) | 14051 (89.6) | 161779 (61.0) | 3205 (94.4) | | 144919 (64.3) | 18785 (91.0) | 14652 (93.4) | 181561 (68.5) |  |  |
| N03 Anti-epileptics | 550 (16.2) | 20166 (8.9) | 4252 (20.6) | 5308 (33.8) | 30276 (11.4) | 1107 (32.6) | | 31894 (14.2) | 7474 (36.2) | 6958 (44.4) | 47433 (17.9) |  |  |
| N05 Psycholeptics | 1746 (51.4) | 87757 (38.9) | 11693 (56.7) | 11274 (71.9) | 112470 (42.4) | 2927 (86.2) | | 103292 (45.8) | 15481 (75.0) | 12458 (79.4) | 134158 (50.6) |  |  |
| N06A Antidepressants | 1623 (47.8) | 76877 (34.1) | 11154 (54.0) | 10986 (70.1) | 100640 (38.0) | 2158 (63.5) | | 92516 (41) | 13916 (67.4) | 11952 (76.2) | 120542 (45.5) |  |  |
| N06B Psychostimulants | 6 (0.2) | 111 (0) | 22 (0.1) | 28 (0.2) | 167 (0.1) | 8 (0.2) | | 134 (0.1) | 26 (0.1) | 29 (0.2) | 197 (0.1) |  |  |
| R03 Drugs for obstructive airway disease | 1834 (54.0) | 90238 (40.0) | 12329 (59.7) | 11438 (72.9) | 115839 (43.7) | 2151 (63.3) | | 107380 (47.6) | 14584 (70.7) | 12244 (78.1) | 136359 (51.4) |  |  |

Figure S2 (a) Intercepts and slopes of ingredients, and (b) Survival probabilities of each cluster in IPCI


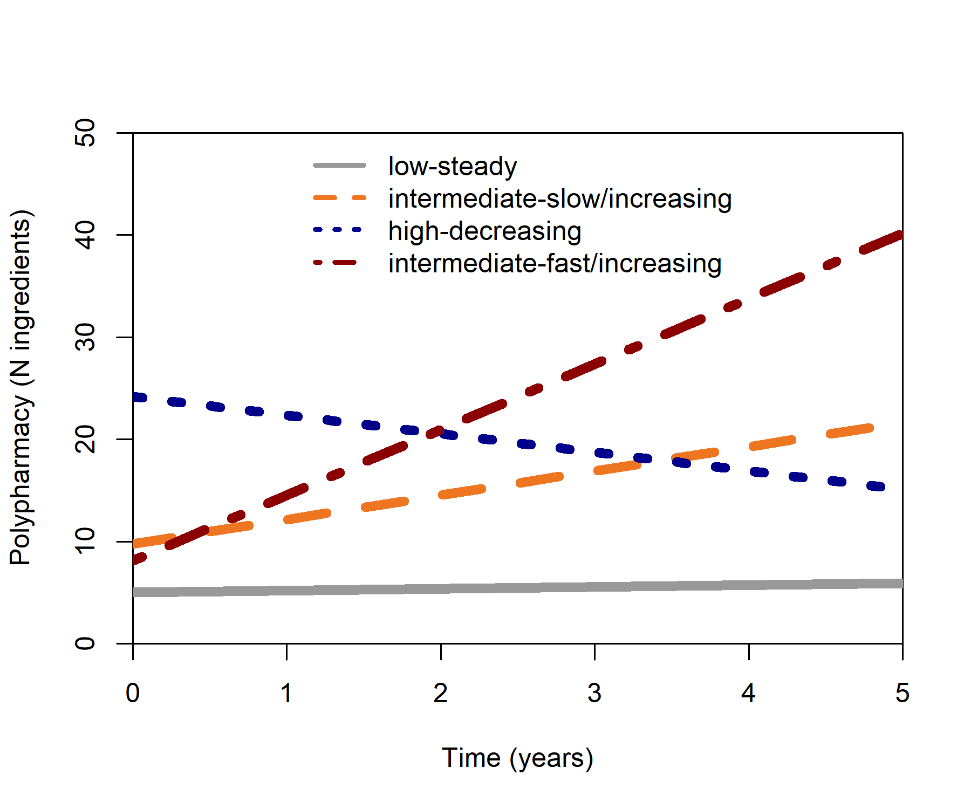


(a)


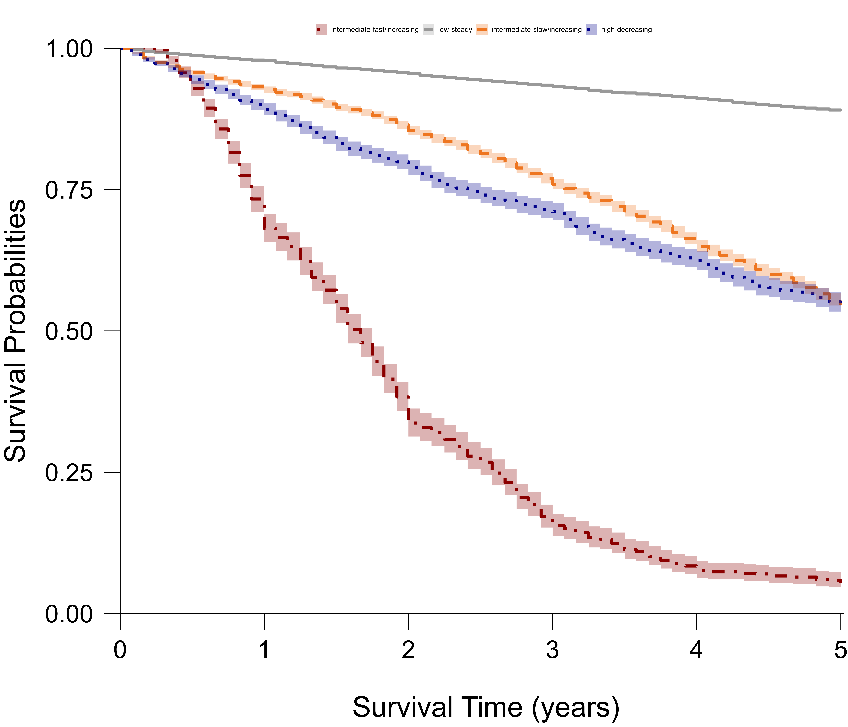


(b)

Table S9 Individual comorbidities at baseline and the last observation in IPCI

|  | At baseline | | | | | | At the last observation | | | | | |
| --- | --- | --- | --- | --- | --- | --- | --- | --- | --- | --- | --- | --- |
|  | Intermediate-fast/increasing  N=1429 | Low-steady N=125835 | Intermediate-slow/increasing N=8268 | High-decreasing N=3775 | Overall N=139307 | Intermediate-fast/increasing  N=1429 | | Low-steady N=125835 | Intermediate-slow/increasing N=8268 | High-decreasing N=3775 | Overall N=139307 |  |
| Mean (SD) | | | | | | | | | | | | |
| Charlson morbidity index | 2.17 (1.71) | 1.16 (1.39) | 1.99 (1.67) | 2.64 (1.90) | 1.26 (1.46) | 3.29 (1.71) | | 1.71 (1.67) | 3.17 (1.89) | 3.46 (2.12) | 1.86 (1.76) |  |
| n (%) | | | | | | | | | | | | |
| Myocardial infarction | 162 (11.3) | 8536 (6.8) | 1039 (12.6) | 598 (15.8) | 10335 (7.4) | 205 (14.3) | | 10565 (8.4) | 1451 (17.5) | 680 (18.0) | 12901 (9.3) |  |
| Congestive heart failure | 188 (13.2) | 6630 (5.3) | 1125 (13.6) | 905 (24.0) | 8848 (6.4) | 350 (24.5) | | 11001 (8.7) | 2219 (26.8) | 1231 (32.6) | 14801 (10.6) |  |
| Peripheral vascular disease | 132 (9.2) | 4831 (3.8) | 727 (8.8) | 492 (13.0) | 6182 (4.4) | 171 (12.0) | | 7555 (6.0) | 1178 (14.2) | 686 (18.2) | 9590 (6.9) |  |
| Cerebrovascular disease | 225 (15.7) | 12320 (9.8) | 1300 (15.7) | 776 (20.6) | 14621 (10.5) | 315 (22.0) | | 18252 (14.5) | 2045 (24.7) | 1003 (26.6) | 21615 (15.5) |  |
| Dementia | 35 (2.4) | 3871 (3.1) | 236 (2.9) | 159 (4.2) | 4301 (3.1) | 81 (5.7) | | 9022 (7.2) | 624 (7.5) | 380 (10.1) | 10107 (7.3) |  |
| Chronic obstructive pulmonary disease | 377 (26.4) | 15033 (11.9) | 2349 (28.4) | 1485 (39.3) | 19244 (13.8) | 432 (30.2) | | 17448 (13.9) | 2786 (33.7) | 1602 (42.4) | 22268 (16.0) |  |
| Rheumatologic disease | 104 (7.3) | 6353 (5.0) | 711 (8.6) | 407 (10.8) | 7575 (5.4) | 120 (8.4) | | 8106 (6.4) | 940 (11.4) | 456 (12.1) | 9622 (6.9) |  |
| Peptic ulcer disease | 84 (5.9) | 4104 (3.3) | 481 (5.8) | 267 (7.1) | 4936 (3.5) | 101 (7.1) | | 4704 (3.7) | 552 (6.7) | 299 (7.9) | 5656 (4.1) |  |
| Mild liver disease | 14 (1.0) | 314 (0.2) | 43 (0.5) | 37 (1.0) | 408 (0.3) | 22 (1.5) | | 422 (0.3) | 73 (0.9) | 46 (1.2) | 563 (0.4) |  |
| Diabetes with chronic complications | 49 (3.4) | 2378 (1.9) | 367 (4.4) | 288 (7.6) | 3082 (2.2) | 67 (4.7) | | 3478 (2.8) | 529 (6.4) | 386 (10.2) | 4460 (3.2) |  |
| Hemiplegia or paraplegia | 0 (0) | 0 (0) | 0 (0) | 0 (0) | 0 (0) | 0 (0) | | 0 (0) | 0 (0) | 0 (0) | 0 (0) |  |
| Renal disease | 200 (14.0) | 8327 (6.6) | 1097 (13.3) | 726 (19.2) | 10350 (7.4) | 305 (21.3) | | 18418 (14.6) | 2318 (28.0) | 1208 (32.0) | 22249 (16.0) |  |
| Any malignancy | 632 (44.2) | 30752 (24.4) | 2755 (33.3) | 1386 (36.7) | 35525 (25.5) | 1069 (74.8) | | 42033 (33.4) | 4315 (52.2) | 1706 (45.2) | 49123 (35.3) |  |
| Moderate to severe liver disease | 4 (0.3) | 37 (0) | 6 (0.1) | 9 (0.2) | 56 (0) | 6 (0.4) | | 51 (0) | 11 (0.1) | 12 (0.3) | 80 (0.1) |  |
| Metastatic solid tumour | 0 (0) | 0 (0) | 0 (0) | 0 (0) | 0 (0) | 0 (0) | | 0 (0) | 0 (0) | 0 (0) | 0 (0) |  |
| AIDS | 0 (0) | 60 (0) | <5 | <5 | 67 (0) | 0 (0) | | 66 (0.1) | <5 | <5 | 74 (0.1) |  |
| Hypertension | 492 (34.4) | 44158 (35.1) | 3008 (36.4) | 1494 (39.6) | 49152 (35.3) | 562 (39.3) | | 55117 (43.8) | 3860 (46.7) | 1745 (46.2) | 61284 (44) |  |
| Heart failure | 134 (9.4) | 4413 (3.5) | 737 (8.9) | 688 (18.2) | 5972 (4.3) | 313 (21.9) | | 9297 (7.4) | 2003 (24.2) | 1070 (28.3) | 12683 (9.1) |  |
| Osteoporosis | 83 (5.8) | 6222 (4.9) | 447 (5.4) | 377 (10.0) | 7129 (5.1) | 122 (8.5) | | 9273 (7.4) | 818 (9.9) | 524 (13.9) | 10737 (7.7) |  |
| Gastroesophageal reflux disease | 15 (1.0) | 1929 (1.5) | 151 (1.8) | 82 (2.2) | 2177 (1.6) | 27 (1.9) | | 2835 (2.3) | 232 (2.8) | 119 (3.2) | 3213 (2.3) |  |
| Chronic kidney disease | 5 (0.3) | 122 (0.1) | 19 (0.2) | 11 (0.3) | 157 (0.1) | 7 (0.5) | | 204 (0.2) | 40 (0.5) | 14 (0.4) | 265 (0.2) |  |
| Venous thromboembolism | 49 (3.4) | 2854 (2.3) | 265 (3.2) | 187 (5.0) | 3355 (2.4) | 140 (9.8) | | 5503 (4.4) | 672 (8.1) | 322 (8.5) | 6637 (4.8) |  |
| Hypothyroidism | 46 (3.2) | 4184 (3.3) | 294 (3.6) | 243 (6.4) | 4767 (3.4) | 73 (5.1) | | 6503 (5.2) | 558 (6.7) | 343 (9.1) | 7477 (5.4) |  |
| Stroke | 73 (5.1) | 4437 (3.5) | 462 (5.6) | 298 (7.9) | 5270 (3.8) | 151 (10.6) | | 9175 (7.3) | 1065 (12.9) | 488 (12.9) | 10879 (7.8) |  |
| Anxiety | 178 (12.5) | 15472 (12.3) | 1120 (13.5) | 790 (20.9) | 17560 (12.6) | 284 (19.9) | | 24162 (19.2) | 2056 (24.9) | 1134 (30.0) | 27636 (19.8) |  |
| Asthma | 69 (4.8) | 5514 (4.4) | 591 (7.1) | 481 (12.7) | 6655 (4.8) | 87 (6.1) | | 7543 (6.0) | 907 (11.0) | 622 (16.5) | 9159 (6.6) |  |
| Pneumonia | 171 (12.0) | 7235 (5.7) | 877 (10.6) | 758 (20.1) | 9041 (6.5) | 419 (29.3) | | 16602 (13.2) | 2521 (30.5) | 1321 (35) | 20863 (15.0) |  |
| Diabetes | 303 (21.2) | 17789 (14.1) | 1971 (23.8) | 1205 (31.9) | 21268 (15.3) | 376 (26.3) | | 22062 (17.5) | 2548 (30.8) | 1401 (37.1) | 26387 (18.9) |  |
| Inflammatory bowel disease | 9 (0.6) | 681 (0.5) | 75 (0.9) | 59 (1.6) | 824 (0.6) | 13 (0.9) | | 991 (0.8) | 118 (1.4) | 77 (2.0) | 1199 (0.9) |  |
| Depressive disorder | 62 (4.3) | 4075 (3.2) | 433 (5.2) | 363 (9.6) | 4933 (3.5) | 112 (7.8) | | 6084 (4.8) | 775 (9.4) | 505 (13.4) | 7476 (5.4) |  |

Table S10 Drug use by drug classes and common drugs at baseline and the last observation for each cluster and the overall population in IPCI

|  | At baseline (n (%)) | | | | | | At the last observation (n (%)) | | | | | |
| --- | --- | --- | --- | --- | --- | --- | --- | --- | --- | --- | --- | --- |
|  | Intermediate-fast/increasing  N=1429 | Low-steady N=125835 | Intermediate-slow/increasing N=8268 | High-decreasing N=3775 | Overall N=139307 | Intermediate-fast/increasing  N=1429 | | Low-steady N=125835 | Intermediate-slow/increasing N=8268 | High-decreasing N=3775 | Overall N=139307 |  |
| Drug classes (ATC name) | | | | | | | | | | | | |
| A: Alimentary tract and metabolism | 1083 (75.8) | 76827 (61.1) | 6943 (84.0) | 3768 (99.8) | 88621 (63.6) | 1426 (99.8) | | 82120 (65.3) | 8211 (99.3) | 3516 (93.1) | 95273 (68.4) |  |
| B: Blood and blood forming organs | 626 (43.8) | 34629 (27.5) | 4027 (48.7) | 2772 (73.4) | 42054 (30.2) | 1021 (71.4) | | 44634 (35.5) | 6202 (75.0) | 2506 (66.4) | 54363 (39.0) |  |
| C: Cardiovascular system | 1065 (74.5) | 80016 (63.6) | 6763 (81.8) | 3595 (95.2) | 91439 (65.6) | 1229 (86.0) | | 83501 (66.4) | 7471 (90.4) | 3258 (86.3) | 95459 (68.5) |  |
| D: Dermatological | 494 (34.6) | 31145 (24.8) | 3489 (42.2) | 2692 (71.3) | 37820 (27.1) | 757 (53.0) | | 28827 (22.9) | 4634 (56.0) | 1931 (51.2) | 36149 (25.9) |  |
| G: Genito-urinary system and sex hormones | 270 (18.9) | 16505 (13.1) | 1755 (21.2) | 1251 (33.1) | 19781 (14.2) | 407 (28.5) | | 16922 (13.4) | 2362 (28.6) | 861 (22.8) | 20552 (14.8) |  |
| H: Systemic hormonal preparations, excluding sex hormones and insulins | 132 (9.2) | 9107 (7.2) | 1001 (12.1) | 898 (23.8) | 11138 (8.0) | 276 (19.3) | | 10134 (8.1) | 1514 (18.3) | 745 (19.7) | 12669 (9.1) |  |
| J: Anti-infective for systemic use | 604 (42.3) | 40099 (31.9) | 3996 (48.3) | 3116 (82.5) | 47815 (34.3) | 1067 (74.7) | | 39626 (31.5) | 5771 (69.8) | 2241 (59.4) | 48705 (35.0) |  |
| L: Antineoplastic and immunomodulation agents | 125 (8.7) | 3942 (3.1) | 599 (7.2) | 491 (13.0) | 5157 (3.7) | 241 (16.9) | | 4864 (3.9) | 1044 (12.6) | 358 (9.5) | 6507 (4.7) |  |
| M: Musculoskeletal system | 393 (27.5) | 28891 (23.0) | 2644 (32.0) | 2033 (53.9) | 33961 (24.4) | 674 (47.2) | | 23802 (18.9) | 3397 (41.1) | 1259 (33.4) | 29132 (20.9) |  |
| N: Nervous system | 657 (46.0) | 41324 (32.8) | 4482 (54.2) | 3249 (86.1) | 49712 (35.7) | 1378 (96.4) | | 44272 (35.2) | 6899 (83.4) | 2900 (76.8) | 55449 (39.8) |  |
| P: Anti-parasitic products, insecticides and repellents | 125 (8.7) | 3942 (3.1) | 599 (7.2) | 491 (13.0) | 5157 (3.7) | 241 (16.9) | | 4864 (3.9) | 1044 (12.6) | 358 (9.5) | 6507 (4.7) |  |
| R: Respiratory system | 539 (37.7) | 29120 (23.1) | 3659 (44.3) | 2744 (72.7) | 36062 (25.9) | 822 (57.5) | | 28370 (22.5) | 4805 (58.1) | 2160 (57.2) | 36157 (26.0) |  |
| S: Sensory organs | 131 (9.2) | 8451 (6. 7) | 921 (11.1) | 848 (22.5) | 10351 (7.4) | 523 (36.6) | | 10736 (8.5) | 1917 (23.2) | 594 (15.7) | 13770 (9.9) |  |
| V: Various | 10 (0.7) | 206 (0.2) | 57 (0.7) | 126 (3.3) | 399 (0.3) | 74 (5.2) | | 286 (0.2) | 165 (2.0) | 88 (2.3) | 613 (0.4) |  |
| Drugs (ATC name) | | | | | | | | | | | | |
| A02 Drugs for acid related disorder | 907 (63.5) | 63406 (50.4) | 5903 (71.4) | 3504 (92.8) | 73720 (52.9) | 1326 (92.8) | | 85197 (67.7) | 7764 (93.9) | 3675 (97.4) | 97962 (70.3) |  |
| A10A/ A10B Drugs used in diabetes | 344 (24.1) | 17305 (13.8) | 2317 (28) | 1488 (39.4) | 21454 (15.4) | 423 (29.6) | | 20416 (16.2) | 2770 (33.5) | 1588 (42.1) | 25197 (18.1) |  |
| B01A Anti-thrombotic agents | 441 (30.9) | 23913 (19.0) | 2721 (32.9) | 1932 (51.2) | 29007 (20.8) | 816 (57.1) | | 40873 (32.5) | 5266 (63.7) | 2435 (64.5) | 49390 (35.5) |  |
| C03 Diuretics | 567 (39.7) | 34210 (27.2) | 3538 (42.8) | 2378 (63.0) | 40693 (29.2) | 876 (61.3) | | 48029 (38.2) | 5575 (67.4) | 2804 (74.3) | 57284 (41.1) |  |
| C07 Beta blocking agents | 606 (42.4) | 41594 (33.1) | 3971 (48.0) | 2336 (61.9) | 48507 (34.8) | 817 (57.2) | | 51339 (40.8) | 5346 (64.7) | 2567 (68) | 60069 (43.1) |  |
| C08 Calcium channel blockers | 370 (25.9) | 23439 (18.6) | 2566 (31.0) | 1595 (42.3) | 27970 (20.1) | 513 (35.9) | | 34352 (27.3) | 3810 (46.1) | 1879 (49.8) | 40554 (29.1) |  |
| C09 Agents renin-angiotensin systemic | 733 (51.3) | 50313 (40.0) | 4785 (57.9) | 2618 (69.4) | 58449 (42.0) | 840 (58.8) | | 60891 (48.4) | 5834 (70.6) | 2804 (74.3) | 70369 (50.5) |  |
| C10 Lipid modifying agents | 727 (50.9) | 49930 (39.7) | 4732 (57.2) | 2607 (69.1) | 57996 (41.6) | 836 (58.5) | | 60585 (48.1) | 5749 (69.5) | 2769 (73.4) | 69939 (50.2) |  |
| G03A Hormonal contraceptives for systemic use | <5 | 163 (0.1) | 22 (0.3) | 22 (0.6) | 209 (0.2) | 5 (0.3) | | 228 (0.2) | 37 (0.4) | 23 (0.6) | 293 (0.2) |  |
| J01 Antibacterial systemic | 844 (59.1) | 60194 (47.8) | 5260 (63.6) | 3273 (86.7) | 69571 (49.9) | 1264 (88.5) | | 88149 (70.1) | 7631 (92.3) | 3632 (96.2) | 100676 (72.3) |  |
| L01 Antineoplastic agents | 87 (6.1) | 4034 (3.2) | 551 (6.7) | 386 (10.2) | 5058 (3.6) | 208 (14.6) | | 6723 (5.3) | 1144 (13.8) | 521 (13.8) | 8596 (6.2) |  |
| L04A Immunosuppressant | 41 (2.9) | 1684 (1.3) | 288 (3.5) | 234 (6.2) | 2247 (1.6) | 75 (5.2) | | 2467 (2.0) | 546 (6.6) | 296 (7.8) | 3384 (2.4) |  |
| M01A/ H02 Anti-inflammatory and/or anti-rheumatic | 737 (51.6) | 55675 (44.2) | 4781 (57.8) | 2956 (78.3) | 64149 (46.0) | 1208 (84.5) | | 77965 (62.0) | 6942 (84.0) | 3330 (88.2) | 89445 (64.2) |  |
| N02A Opioids | 451 (31.6) | 25385 (20.2) | 3111 (37.6) | 2520 (66.8) | 31467 (22.6) | 1286 (90.0) | | 43528 (34.6) | 6343 (76.7) | 3108 (82.3) | 54265 (39.0) |  |
| N03 Anti-epileptics | 136 (9.5) | 5920 (4.7) | 872 (10.5) | 846 (22.4) | 7774 (5.6) | 349 (24.4) | | 10504 (8.3) | 1973 (23.9) | 1208 (32) | 14034 (10.1) |  |
| N05 Psycholeptics | 486 (34.0) | 32698 (26.0) | 3315 (40.1) | 2419 (64.1) | 38918 (27.9) | 1243 (87.0) | | 45879 (36.5) | 5784 (70..0) | 2874 (76.1) | 55780 (40.0) |  |
| N06A Antidepressants | 226 (15.8) | 13396 (10.6) | 1645 (19.9) | 1356 (35.9) | 16623 (11.9) | 466 (32.6) | | 19815 (15.7) | 2849 (34.5) | 1747 (46.3) | 24877 (17.9) |  |
| N06B Psychostimulants | 6 (0.4) | 173 (0.1) | 19 (0.2) | 28 (0.7) | 226 (0.2) | 32 (2.2) | | 272 (0.2) | 53 (0.6) | 46 (1.2) | 403 (0.3) |  |
| R03 Drugs for obstructive airway disease | 563 (39.4) | 32776 (26.0) | 3804 (46.0) | 2482 (65.7) | 39625 (28.4) | 799 (55.9) | | 46541 (37.0) | 5368 (64.9) | 2801 (74.2) | 55509 (39.8) |  |
